# Association of testosterone and sex hormone–binding globulin with chronic liver disease outcomes across sex and menopausal status

**DOI:** 10.1186/s13293-026-00923-8

**Published:** 2026-05-21

**Authors:** Luna Liu, Wenhui Liu, Yingzhou Shi, Xiaoran Cha, Panpan Yin, Qingbo Guan, Xiude Fan, Mo Wang

**Affiliations:** 1 Department of Vascular Surgery, Shandong Provincial Hospital Affiliated to Shandong First Medical University, Jinan, 250021 Shandong China; 2 Key Laboratory of Endocrine Glucose & Lipids Metabolism and Brain Aging, Department of endocrinology, Ministry of Education, Shandong Provincial Hospital, Shandong First Medical University, Jinan, 250021 Shandong China; 3 Shandong Clinical Research Center of Diabetes and Metabolic Diseases, Jinan, 250021 Shandong China; 4 College of Clinical Medicine, Shandong Second Medical University, Weifang, Shandong China; 5 College of Clinical Medicine and Basic Medical Sciences, Shandong first Medical University, Jinan, China; 6 Department of ultrasound, Shandong Provincial Hospital Affiliated to Shandong First Medical University, Jinan, 250021 Shandong China

**Keywords:** Chronic Liver Disease, Total testosterone, Sex hormone-binding globulin, Sex, Liver fibrosis and cirrhosis

## Abstract

**Background & Aims:**

The influence of sex hormones on chronic liver disease (CLD) progression remains unclear, despite sex-related differences in disease susceptibility and outcomes. This study aimed to investigate the associations of total testosterone (TT) and sex hormone-binding globulin (SHBG) levels with CLD-related outcomes across sex and menopausal status.

**Methods:**

A retrospective cohort study was conducted using UK Biobank data. Serum TT and SHBG levels were measured at baseline. Participants were followed for incident liver fibrosis and cirrhosis, hepatocellular carcinoma (HCC), and liver-related death. Cumulative risk curve, restricted cubic spline, and multivariable Cox proportional hazards models were used to assess TT and SHBG associations with outcomes in males, premenopausal females, and postmenopausal females.

**Results:**

A total of 155,997 males, 79,637 postmenopausal females, and 28,174 premenopausal females were included, with a median follow-up of 13.53 years. Cumulative risk curve showed that males had the highest risk of CLD-related outcomes (*P* < 0.001). Males in the highest quartile of SHBG had hazard ratios (HRs) of 3.58 for liver fibrosis and cirrhosis, 4.31 for HCC, and 5.18 for liver-related death compared to the lowest quartile. In the highest TT quartile, the HRs were 2.17 for liver fibrosis and cirrhosis, 2.22 for HCC, and 2.50 for liver-related death. In postmenopausal females, only SHBG levels were positively associated with outcomes, though weaker than males. No significant associations were observed in premenopausal females.

**Conclusions:**

Elevated TT and SHBG levels are associated with higher risks of CLD-related outcomes, particularly in males. Further studies are needed to clarify the causal roles and mechanisms.

**Graphical Abstract:**

**Figure:**
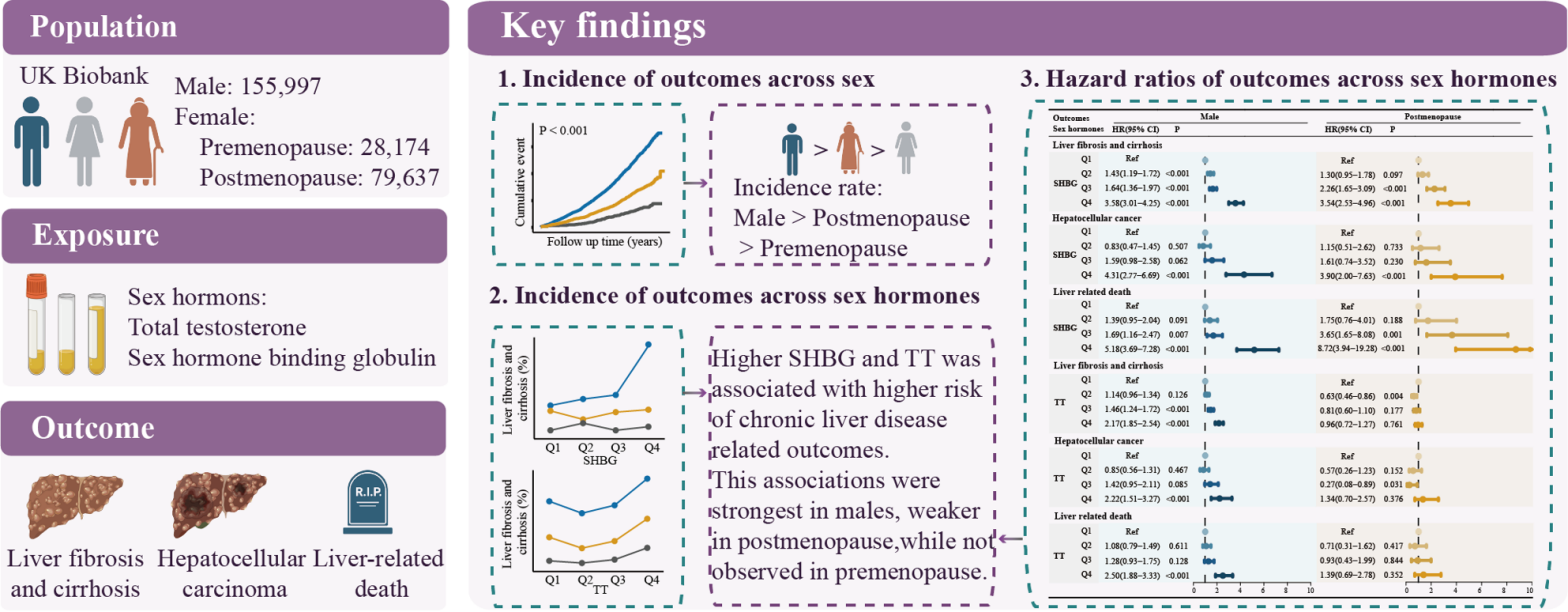

**Supplementary Information:**

The online version contains supplementary material available at 10.1186/s13293-026-00923-8.

## Abstract (plain language summary)

This study looked at how the body’s main male hormone (testosterone) and a related carrier protein (SHBG) are linked to long-term liver disease. Using health data from over 260,000 people in the UK, we followed them for more than 13 years to see who developed liver fibrosis, liver cancer, or died from liver disease. We found that men with higher testosterone and higher SHBG levels were more likely to later develop serious liver problems. In women, the same pattern was seen mostly after menopause, but the link was weaker. In younger women before menopause, we didn’t see a clear connection. These findings suggest that hormonal differences between men and women may partly explain why men tend to get more severe liver disease and liver cancer. Understanding this may help doctors recognize who is at higher risk and could lead to new ways to prevent or monitor liver disease in people with certain hormone patterns.

**Supplementary Information:**

The online version contains supplementary material available at 10.1186/s13293-026-00923-8.

## Highlights

- In a UK population over 260,000 people, higher testosterone and SHBG levels were associated with greater long-term risk of liver fibrosis, liver cancer, and liver-related death.
- The strongest associations were seen in men; postmenopausal women showed weaker but still present links. No clear associations were detected in premenopausal women.
- Results suggest sex-related hormone differences may help explain why men have worse liver disease outcomes.
- Findings support further research into hormone-related pathways as potential targets for liver disease prevention and monitoring.

**Supplementary Information:**

The online version contains supplementary material available at 10.1186/s13293-026-00923-8.

## Introduction

Chronic liver disease (CLD) represents a major global public health burden and remains a leading cause of morbidity and mortality worldwide [1–3]. CLD usually arises from sustained hepatic injury of diverse etiologies, including chronic viral hepatitis, excessive alcohol consumption, metabolic dysfunction-associated steatotic liver disease (MASLD), and autoimmune liver diseases [4, 5]. Persistent liver injury can drive progressive fibrosis, eventually leading to cirrhosis and hepatocellular carcinoma (HCC) [6], which together contribute substantially to liver-related mortality and healthcare burden. Recent estimates indicate that CLD accounted for approximately 58 million incident cases and over 1.4 million deaths [7], underscoring their growing global impact. Moreover, about two-thirds of liver-related deaths occur among males [1], suggesting that biological sex may play a crucial role in disease susceptibility and progression, which warrants further investigation.

Accumulating evidence highlights the significant role of sex differences in the etiology, progression, and outcomes of CLD [8, 9]. Men are more frequently affected by alcohol-related liver disease (ALD), as they tend to drink more frequently and in larger quantities [10]. However, epidemiological data suggest that when consuming equal amounts of alcohol, women are more likely to develop ALD, with faster progression and worse prognosis [11]. Women also exhibit a higher prevalence of autoimmune liver diseases, including autoimmune hepatitis and primary biliary cholangitis [12, 13], which show a strong female predominance, whereas primary sclerosing cholangitis is more common in men and is associated with poorer survival outcomes [14]. In MASLD, men generally have a higher prevalence, although the risk for women increases substantially after menopause [15–17]. Males exhibit more rapid progression of liver fibrosis and worse overall outcomes than females, with the global death rate estimated to be 1.5 times higher in men [18], and the incidence of hepatocellular carcinoma three to four times greater than in women [19, 20]. Although the etiological spectrum of CLD differs between men and women, these differences alone cannot fully account for the marked sex-related disparities in disease progression and outcomes.

Although the role of androgens in CLD has attracted considerable research attention, their relationship with disease outcomes remains controversial and poorly defined. Previous studies showed that men with cirrhosis frequently exhibited hypogonadism [21, 22], and lower total or free testosterone has been linked to higher risks of major infection, liver transplantation, and death [23], suggesting prognostic value for low testosterone in advanced liver disease. Conversely, a study found that higher circulating testosterone was associated with advanced fibrosis and inflammatory activity in males with hepatitis C virus infection [24], implying a potential pro-fibrotic role of androgen signaling during progression toward cirrhosis. Epidemiological data regarding HCC are relatively consistent, showing that elevated levels of testosterone and SHBG are generally associated with an increased incidence of HCC and worse clinical outcomes in males and postmenopausal females [25–27]. Mechanistically, experimental data indicate that androgen signaling can aggravate hepatic fibrosis by enhancing NLRP3 inflammasome activation [28], and that androgens further promote hepatocarcinogenesis by acting on androgen-response elements within the viral genome and engaging multiple oncogenic pathways [29–31]. These data underscore a complex and only partially understood role of androgen signaling in the progression of CLD, as associations with clinical outcomes in men are inconsistent across studies, whereas in women the impact of androgens remains largely unexplored because of a paucity of dedicated research.

In this retrospective cohort study using data from the UK Biobank (UKB), we aimed to investigate the associations of serum total testosterone (TT) and sex hormone-binding globulin (SHBG) levels with CLD-related outcomes, including liver fibrosis and cirrhosis, HCC, and liver-related death, separately in male, premenopausal female, and postmenopausal female participants.

## Patients and methods

### Study design and participants

This study used data from the UKB, a nationwide resource of more than 500,000 participants aged 37–73 years who were enrolled between 2006 and 2010 at 22 assessment centres across the United Kingdom. At the initial assessment, information on sociodemographic profile, lifestyle behaviours, and medical history was obtained through standardized questionnaires and interviews, and participants also underwent physical measurements and blood sampling, allowing the assessment of routine biochemical markers and circulating sex hormones. Health outcomes have since been ascertained through regular linkage of the cohort to national hospital admission records and death registries, providing long-term follow-up of disease events and mortality. The UKB operates under ethical approval from the appropriate research ethics committees, and the present analysis was conducted within the general approval framework of the NHS National Research Ethics Service.

**Fig. 1:**
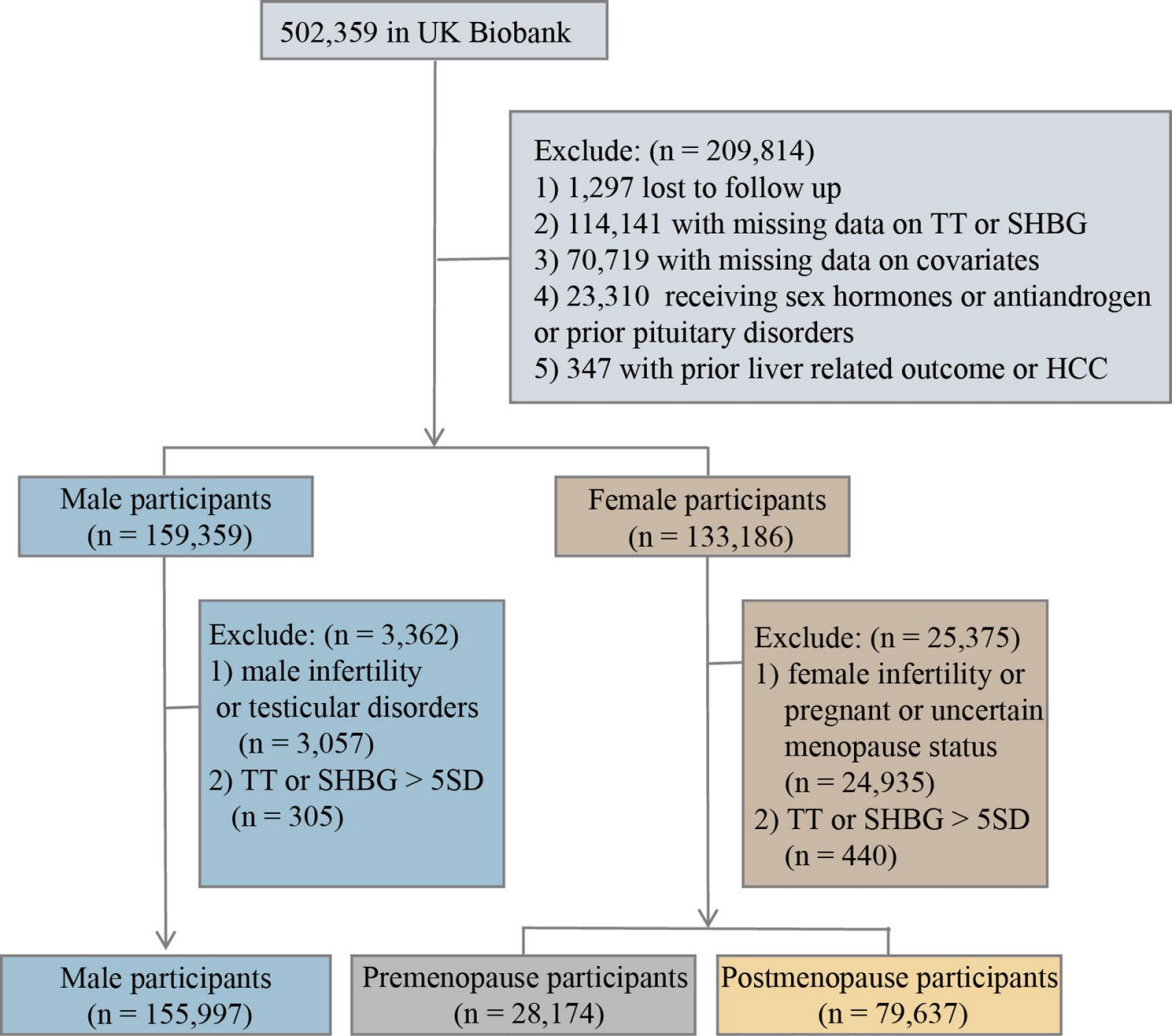
Flowchart. Covariates included age, sex, race, smoking status, drinking status, alcohol intake per day, townsend deprivation index, Qualifications, body mass index, ALT, AST, ALB, TBIL, Uric, Crea, FIB4. TT: total testosterone; SHBG: sex hormone-binding globulin; HCC: hepatocellular carcinoma; SD: standard deviation

We excluded participants who were lost to follow-up (*n* = 1,297), had missing data on TT or SHBG (*n* = 114,141), or lacked information on covariates (*n* = 70,719). Individuals who reported the use of sex hormones or antiandrogen medications, had a history of pituitary disorders (*n* = 23,310), or had a prior diagnosis of liver fibrosis and cirrhosis or HCC (*n* = 347) were also excluded, leaving 292,545 participants eligible for the current analysis. Of these, men with infertility or testicular disorders (*n* = 3,057) or extremely high TT or SHBG concentrations (> 5 SD above the mean, *n* = 305) were excluded, yielding 155,997 male participants. Among women (*n* = 133,186), those with infertility, pregnancy, or uncertain menopausal status (including missing information on menopausal status, not sure about menopausal status due to hysterectomy or other reason, irregular menstrual cycle (*n* = 24,935), or TT/SHBG concentrations > 5 SD above the mean (*n* = 440) were excluded, resulting in 28,174 premenopausal and 79,637 postmenopausal female participants for analysis (Fig. 1).

### Exposures

At baseline, blood samples were collected and processed centrally under standardized protocols, transported in temperature-controlled containers (approximately 4 °C), and stored at − 80 °C until analysis. Serum concentrations of TT and SHBG were quantified using chemiluminescent immunoassays (Beckman Coulter, UK). The analytical range for TT was 0.35–55.52 nmol/L, with coefficients of variation of 8.34%, 3.66%, and 4.15% for low, medium, and high concentrations, respectively. SHBG was measured using a two-step sandwich chemiluminescent immunoassay with an analytical range of 0.33–242 nmol/L, and corresponding coefficients of variation of 5.67%, 5.25%, and 5.22%. Both assays were conducted in the central UK Biobank laboratory using standardized procedures to ensure measurement reliability.

### Outcome and covariates

The outcomes involved in this study included liver fibrosis and cirrhosis, HCC, and liver-related death. Disease diagnoses were ascertained through linkage with hospital inpatient records using the International Classification of Diseases, 10th Revision (ICD-10). Liver fibrosis and cirrhosis were identified by codes K702, K703, K704, K740, K741, K742, K746, K766, I850, and I859, whereas HCC was defined by code C220 [32]. Mortality data were obtained from the National Death Registries, and liver-related deaths were defined by codes K70-K77, I850, I859, and C220. Participants were followed from the date of recruitment until the occurrence of the first outcome event, death, or the end of follow-up on October 31, 2022. Individuals with a history of liver fibrosis, cirrhosis, or HCC at baseline were excluded from the analysis.

Covariates included ‌sociodemographic, lifestyle, baseline biochemical indicators, and clinical factors that may confound the associations of sex hormones with liver disease outcomes. ‌Sociodemographic variables comprised age, race (classified as White or non-White), educational attainment (qualifications) and the Townsend Deprivation Index (TDI), which was categorized into tertiles to indicate low, medium, and high levels of deprivation. Lifestyle factors included smoking status (classified as never, ever, current), alcohol intake frequency (classified as never or special occasions, monthly to weekly, daily or almost daily), alcohol intake per day (g), body mass index (BMI) status (classified as normal (18.5–24.99), underweight (< 18.5), overweight (25.0–29.99), obesity (> = 30.0)) and four indicators of behaviors (classified as unhealthy, healthy, missing), physical activity, diet, sleep, and sedentary behavior, which were defined according to prior studies [33]. Baseline biochemical indicators included alanine aminotransferase (ALT), aspartate aminotransferase (AST), albumin (ALB), total bilirubin (TBIL), uric acid (Uric), creatinine (Crea), fibrosis-4 index (FIB4). Clinical covariates encompassed comorbidity (type 2 diabetes, hypertension, hyperlipidemia, ischemic heart disease, heart failure, stroke, renal failure), current use of relevant medications, including steroids, antihypertensive drugs, antidiabetic agents, and lipid-lowering therapies for cholesterol or triglycerides. Baseline liver diseases included MASLD, metabolic dysfunction-associated steatotic liver disease with increased alcohol intake (MetALD), ALD and viral hepatitis. MASLD was defined as a Fatty Liver Index (FLI) ≥ 60 combined with at least one cardiometabolic risk factor (overweight, hypertension, high diabetes, high triglycerides, low high density lipoprotein), with alcohol consumption not exceeding 210 g/week in males and 140 g/week in females. MetALD was defined as a FLI ≥ 60 combined with at least one cardiometabolic risk factor, with alcohol consumption between 210 and 420 g/week in males and 140–350 g/week in females. ALD was defined as a FLI ≥ 60, with alcohol consumption exceeding 420 g/week in males and 350 g/week in females. Viral hepatitis was definded based on ICD-10 codes (B15-B19).

### Statistical analysis

Continuous variables were summarized as means ± standard deviations, and categorical variables were expressed as percentages. Group differences in continuous and categorical variables were examined using the t-test and chi-square test, respectively. Cumulative incidence curves were generated to compare the risks of liver fibrosis and cirrhosis, HCC, and liver-related death among men, premenopausal women, and postmenopausal women. Given the substantial differences in hormone levels and event rates between these groups, all subsequent analyses were conducted separately for men, premenopausal women, and postmenopausal women. Serum TT and SHBG concentrations were divided into quartiles across sex, and incidence rates of the three outcomes were compared across quartile groups. To explore potential nonlinear associations of TT and SHBG with each liver-related outcome, restricted cubic spline (RCS) models were fitted. Multivariable Cox proportional hazards regression was used to estimate hazard ratios (HRs) and 95% confidence intervals (CIs) for the associations of TT and SHBG quartiles with each outcome. Adjusting covariates including age, race, qualifications, TDI, smoking status, alcohol intake frequency, alcohol intake per day, BMI status, physical activity, diet status, sleep status, and sedentary status, ALT, AST, ALB, TBIL, Uric, Crea, FIB4, baseline liver diseases (MASLD, MetALD, ALD, viral hepatitis), individual binary covariates for comorbid conditions, including type 2 diabetes, hypertension, hyperlipidemia, ischemic heart disease, heart failure, stroke, and renal failure, as well as individual binary covariates for medication use, including steroids, antihypertensive drugs, antidiabetic agents, and lipid-lowering therapies for cholesterol or triglycerides.

The proportional hazards assumption for Cox models was tested, and analyses that violated this assumption were addressed through time-stratified sensitivity analyses. Additional sensitivity analyses were conducted using competing risks regression to account for the competing risk of death, and mutual adjustment of SHBG and TT was performed. Subgroup analyses stratified by age (< 65, >=65), BMI status (normal, overweight or obesity), and the presence of baseline liver disease were performed to evaluate the associations between hormonal status and liver outcomes. All statistical analyses were performed using R software (version 4.3.2). A two-sided p value < 0.05 was considered statistically significant.

## Results

### Baseline characteristics

The study included 155,997 males (mean age: 56.83 years, median follow-up: 13.53 years), 28,174 premenopausal women (mean age: 45.87 years, median follow-up: 13.62 years), and 79,637 postmenopausal women (mean age: 60.41 years, median follow-up: 13.51 years). Most participants were of White ethnicity (94.8% of males, 91.0% of premenopausal women, and 95.7% of postmenopausal women). The mean SHBG levels were highest in premenopausal women (67.56 ± 30.81 nmol/L), followed by postmenopausal women (58.12 ± 26.10 nmol/L) and males (39.38 ± 16.00 nmol/L). The mean TT levels were highest in males (11.92 ± 3.59 nmol/L), followed by premenopausal women (1.21 ± 0.53 nmol/L) and postmenopausal women (1.08 ± 0.52 nmol/L). In terms of baseline liver diseases, MASLD (36.1%), MetALD (13.0%) and ALD (4.4%) were most prevalent in males. Medication use also varied, with antihypertensive drugs being used by 30.7% of males, 9.6% of premenopausal women, and 29.3% of postmenopausal women. Other lifestyle factors, comorbidities, and medication use were detailed in Table 1. Detailed baseline characteristics across quartiles of TT and SHBG can be found in Supplementary Tables 1–6.

**Table 1:** Baseline characteristics

	Male	Premenopause	Postmenopause
*N*	155,997	28,174	79,637
**‌Sociodemographic factors**			
Age (years)	56.83(8.13)	45.87(4.17)	60.41(5.38)
Race (white, %)	147,926(94.8)	25,652(91.0)	76,175(95.7)
Qualifications (%)			
College or University	54,074(34.7)	12,055(42.8)	23,346(29.3)
A levels/AS levels	16,274(10.4)	4187(14.9)	8697(10.9)
O levels/GCSEs	29,549(18.9)	6470(23.0)	18,683(23.5)
CSEs	8551(5.5)	2274(8.1)	3231(4.1)
NVQ or HND or HNC	14,310(9.2)	1020(3.6)	3584(4.5)
Other professional qualifications	7008(4.5)	899(3.2)	5402(6.8)
None of the above	26,231(16.8)	1269(4.5)	16,694(21.0)
TDI (%)			
Tertile 1	52,999(34.0)	8591(30.5)	27,695(34.8)
Tertile 2	51,920(33.3)	9178(32.6)	27,424(34.4)
Tertile 3	51,078(32.7)	10,405(36.9)	24,518(30.8)
**Lifestyle factors**			
Smoking status (%)			
Never	75,730(48.5)	18,168(64.5)	46,586(58.5)
Ever	61,346(39.3)	7122(25.3)	26,783(33.6)
Current	18,921(12.1)	2884(10.2)	6268(7.9)
Alcohol intake per day (g)	22.60(22.23)	11.43(12.88)	10.00(11.74)
Drinking status (%)			
Never or special occasions	22,217(14.2)	6388(22.7)	21,838(27.4)
Monthly to weekly	91,142(58.4)	17,502(62.1)	42,655(53.6)
Daily or almost daily	42,638(27.3)	4284(15.2)	15,144(19.0)
Physical status (%)			
Unhealthy	87,656(56.2)	16,586(58.9)	40,346(50.7)
Healthy	41,312(26.5)	6472(23.0)	18,124(22.8)
Miss	27,029(17.3)	5116(18.2)	21,167(26.6)
Diet status (%)			
Unhealthy	128,676(82.5)	22,905(81.3)	57,548(72.3)
Healthy	25,622(16.4)	5079(18.0)	21,395(26.9)
Miss	1699(1.1)	190(0.7)	694(0.9)
Sleep status (%)			
Unhealthy	18,988(12.2)	2996(10.6)	10,883(13.7)
Healthy	136,499(87.5)	25,074(89.0)	68,263(85.7)
Miss	510(0.3)	104(0.4)	491(0.6)
Sedentary status (%)			
Unhealthy	125,165(80.2)	19,006(67.5)	61,369(77.1)
Healthy	29,909(19.2)	8961(31.8)	17,425(21.9)
Miss	923(0.6)	207(0.7)	843(1.1)
BMI status (%)			
Underweight (< 18.5)	335(0.2)	216(0.8)	547(0.7)
Normal (18.5–24.99)	38,834(24.9)	13,371(47.5)	29,080(36.5)
Overweight (25.0–29.99)	77,712(49.8)	9140(32.4)	30,768(38.6)
Obesity (> = 30.0)	39,116(25.1)	5447(19.3)	19,242(24.2)
**Comorbidity and medications**			
T2DM (%)	11,081(7.1)	512(1.8)	3417(4.3)
Hypertension (%)	126,932(81.4)	12,206(43.3)	59,519(74.7)
Hyperlipidemia (%)	137,410(88.1)	20,187(71.7)	74,665(93.8)
Ischemic heart disease (%)	12,594(8.1)	169(0.6)	2925(3.7)
Heart failure (%)	1292(0.8)	14(0.0)	244(0.3)
Stroke (%)	2990(1.9)	146(0.5)	1067(1.3)
Renal failure (%)	2482(1.6)	88(0.3)	1181(1.5)
Antidiabetic drugs (%)	7954(5.1)	399(1.4)	2390(3.0)
Antihypertensive drugs (%)	47,875(30.7)	2716(9.6)	23,295(29.3)
Antihypertriglyceridemic drugs (%)	1260(0.8)	23(0.1)	309(0.4)
Antihypercholesterolemic drugs (%)	42,808(27.4)	1658(5.9)	18,309(23.0)
Steroids-related drug (%)	7118(4.6)	822(2.9)	3430(4.3)
**Liver diseases and liver status**			
MASLD (%)	56,335(36.1)	3715(13.2)	18,705(23.5)
MetALD (%)	20,226(13.0)	802(2.8)	3010(3.8)
ALD (%)	6806(4.4)	151(0.5)	372(0.5)
Viral hepatitis (%)	169(0.1)	15(0.1)	54(0.1)
ALT (U/L)	27.38(15.03)	17.13(10.25)	21.04(12.04)
AST (U/L)	28.19(10.55)	21.86(7.93)	25.41(9.00)
ALB (g/L)	45.57(2.59)	44.95(2.59)	45.14(2.54)
TBIL (umol/L)	10.32(4.84)	8.51(4.20)	8.20(3.53)
Uric (umol/L)	355.60(71.48)	248.94(57.72)	279.89(66.88)
Crea (umol/L)	81.82(18.37)	63.23(10.54)	64.64(13.76)
FIB4	1.43(2.49)	1.01(3.51)	1.40(1.35)
**Sex hormones**			
SHBG (nmol/L)	39.38(16.00)	67.56(30.81)	58.12(26.10)
TT (nmol/L)	11.92(3.59)	1.21(0.53)	1.08(0.52)
E2 (pmol/L)*	221.83(77.29)	575.41(441.71)	369.72(354.07)
**Follow-up outcomes**			
Liver fibrosis and cirrhosis (%)	1315(0.8)	68(0.2)	345(0.4)
Hepatocellular cancer (%)	212(0.14)	4(0.01)	38(0.05)
Liver related death (%)	393(0.3)	13(0.05)	61(0.08)

TDI: townsend deprivation index; T2DM: type 2 diabetes mellitus; MASLD: Metabolic dysfunction-associated steatotic liver disease; MetALD: Metabolic dysfunction-associated steatotic liver disease with increased alcohol intake; ALD: alcohol-related liver disease; ALT: alanine aminotransferase; AST: aspartate aminotransferase; ALB: albumin; TBIL: total bilirubin; Uric: uric acid; Crea: creatinine; FIB4: fibrosis-4 index; SHBG: sex hormone binding globulin; TT: total testosterone; E2: oestradiol

Continuous variables (age, alcohol intake per day, ALT, AST, ALB, TBIL, Uric, Crea, FIB4, SHBG, TT and E2) are presented as means (standard deviations), and categorical variables are expressed as N (percentages). The t-test was employed to compare differences in continuous variables, while chi-square tests were used for categorical variables

*Among them, E2 was measured in 13,144 men, 19,924 premenopausal women and 2,618 postmenopausal women

### Incidence of CLD-related outcome across sex and sex hormones

**Fig. 2:**
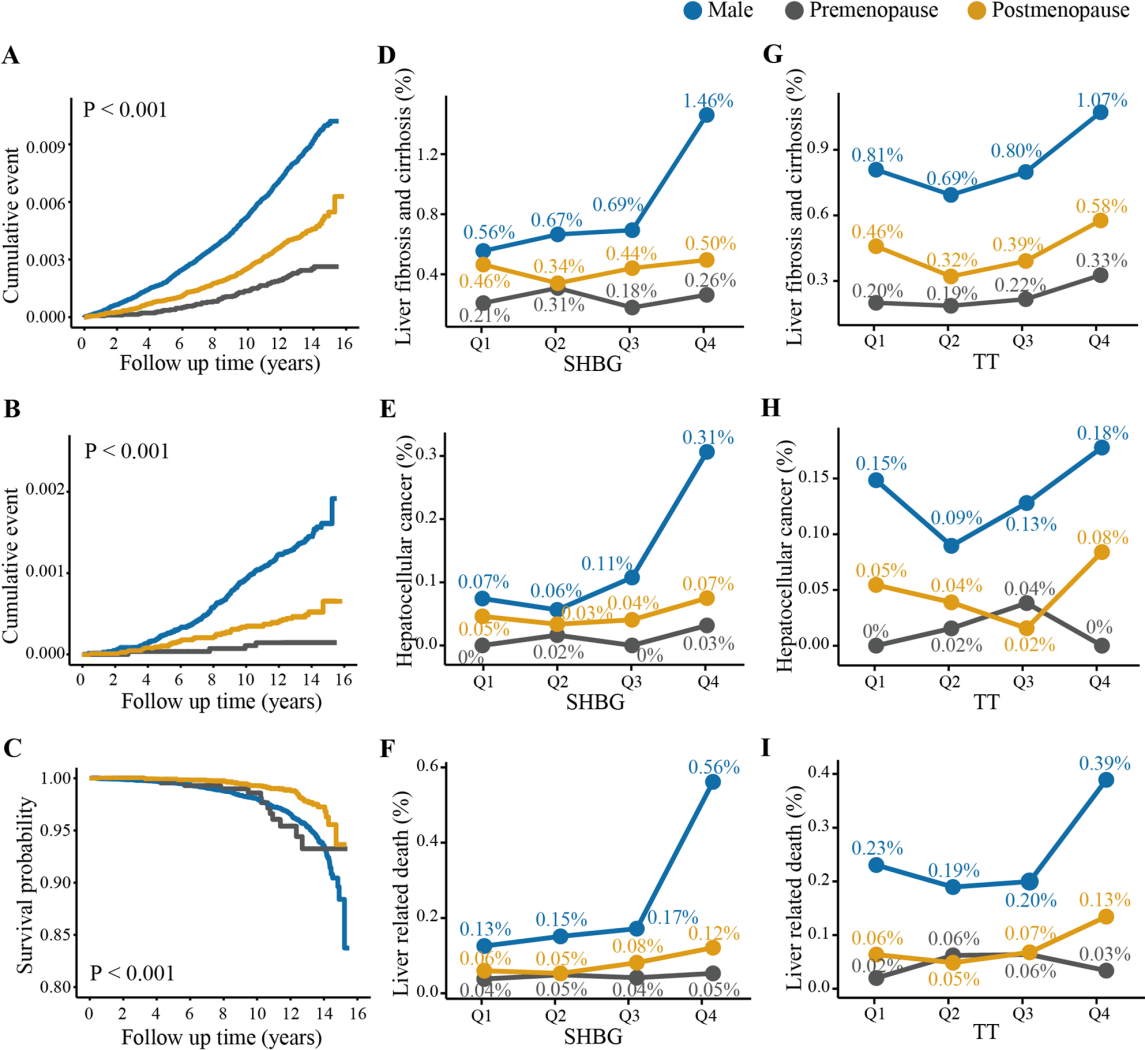
Incidence of chronic liver disease-related outcomes stratified by sex and sex hormone levels. **A**: cumulative risk curve for liver fibrosis and cirrhosis; **B**: cumulative risk curve for hepatocellular cancer; **C**: cumulative risk curve for liver-related death; **D**: incidence of liver fibrosis and cirrhosis across quartiles of SHBG; **E**: incidence of hepatocellular cancer across quartiles of SHBG; **F**: incidence of liver-related death across quartiles of SHBG; **G**: incidence of liver fibrosis and cirrhosis across quartiles of TT; **H**: incidence of hepatocellular cancer across quartiles of TT; **I**: incidence of liver-related death across quartiles of TT. The blue line represents males, the gray line represents premenopausal females, and the yellow line represents postmenopausal females. SHBG: sex hormone-binding globulin; TT: total testosterone; Q1: quartile 1; Q2: quartile 2; Q3: quartile 3; Q4: quartile 4

The incidence rates of liver fibrosis and cirrhosis, HCC, and liver-related death were compared across male, premenopausal female, and postmenopausal female participants. The incidence of liver fibrosis and cirrhosis was highest in males (0.8%), followed by postmenopausal women (0.4%) and premenopausal women (0.2%). HCC was most commonly diagnosed in males (0.14%), with a significantly lower incidence in both premenopausal (0.01%) and postmenopausal women (0.05%). Similarly, liver-related death was more prevalent in males (0.3%) compared to premenopausal (0.05%) and postmenopausal women (0.08%) (Table 1). Cumulative risk curves, as shown in Fig. 2A-C, further demonstrated that males consistently exhibiting higher cumulative risks for all three outcomes (*P* < 0.001). Postmenopausal women showed intermediate risk levels, while premenopausal women had the lowest cumulative risks for liver fibrosis and cirrhosis, HCC, and liver-related death.

To assess the impact of sex hormone levels on CLD-related outcomes, we stratified participants by quartiles of SHBG and TT and compared the incidence of liver fibrosis and cirrhosis, HCC, and liver-related death across different sex groups. For liver fibrosis and cirrhosis, men had higher incidence than postmenopausal women across all SHBG quartiles, whereas premenopausal women consistently showed the lowest rates (Fig. 2D). In men, the incidence rose steeply with increasing SHBG, reaching 1.46% in Q4 compared with 0.56% in Q1, while the corresponding gradients in both female groups were much more modest. When stratified by TT, a similar pattern was observed, while incidence was highest in Q4 and lowest in Q2 (Fig. 2G). Comparable trends were seen for HCC and liver-related death, with males showing the highest absolute risks and the steepest increase across SHBG and TT quartiles (Fig. 2E-F and H-I). These findings highlight the significant association between higher SHBG and TT levels and increased CLD-related outcomes, particularly in males, with females showing a similar but less pronounced pattern.

### RCS analyses of associations between sex hormones and CLD-related outcomes across sex

**Fig. 3:**
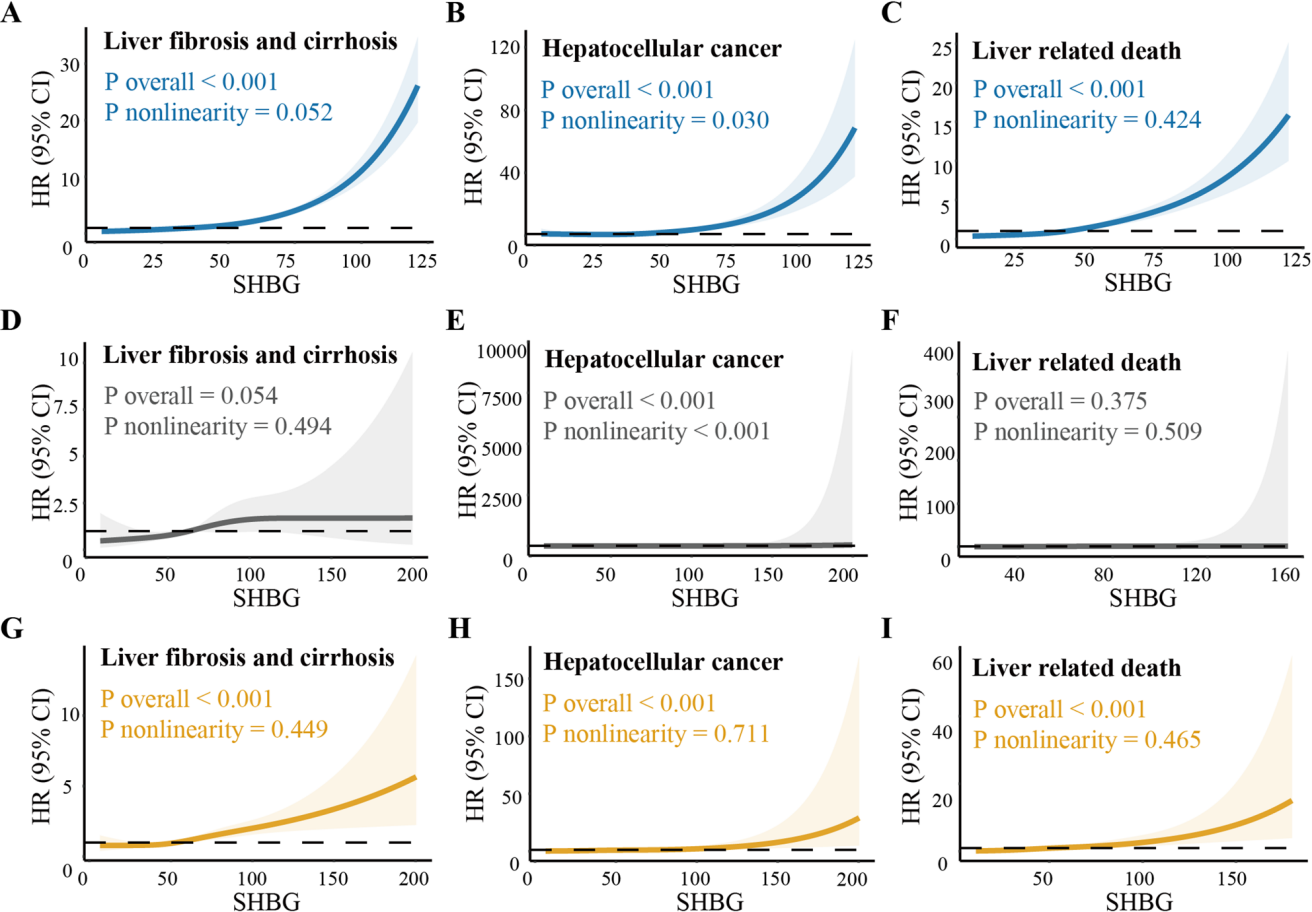
Restricted cubic spline analyses of the association between SHBG and chronic liver disease-related outcomes. **A**: for liver fibrosis and cirrhosis in males; **B**: for hepatocellular cancer in males; **C**: for liver related death in males; **D**: for liver fibrosis and cirrhosis in premenopausal females; **E**: for hepatocellular cancer in premenopausal females; **F**: for liver related death in premenopausal females; **G**: for liver fibrosis and cirrhosis in postmenopausal females; **H**: for hepatocellular cancer in postmenopausal females; **I**: for liver related death in postmenopausal females; Adjusted for age, race, qualifications, townsend deprivation index, smoking status, alcohol intake frequency, BMI status, physical activity, diet status, sleep status, and sedentary status, comorbidity (hypertension, diabetes, hyperlipidemia, ischemic heart disease, heart failure, stroke, renal failure) and medications (antihypertensive drugs, antidiabetic drugs, antihypertriglyceridemic drugs, antihypercholesterolemic drugs, steroids-related drug). SHBG: sex hormone-binding globulin; HR: hazard ratios; CI: confidence interval; BMI: body mass index

**Fig. 4:**
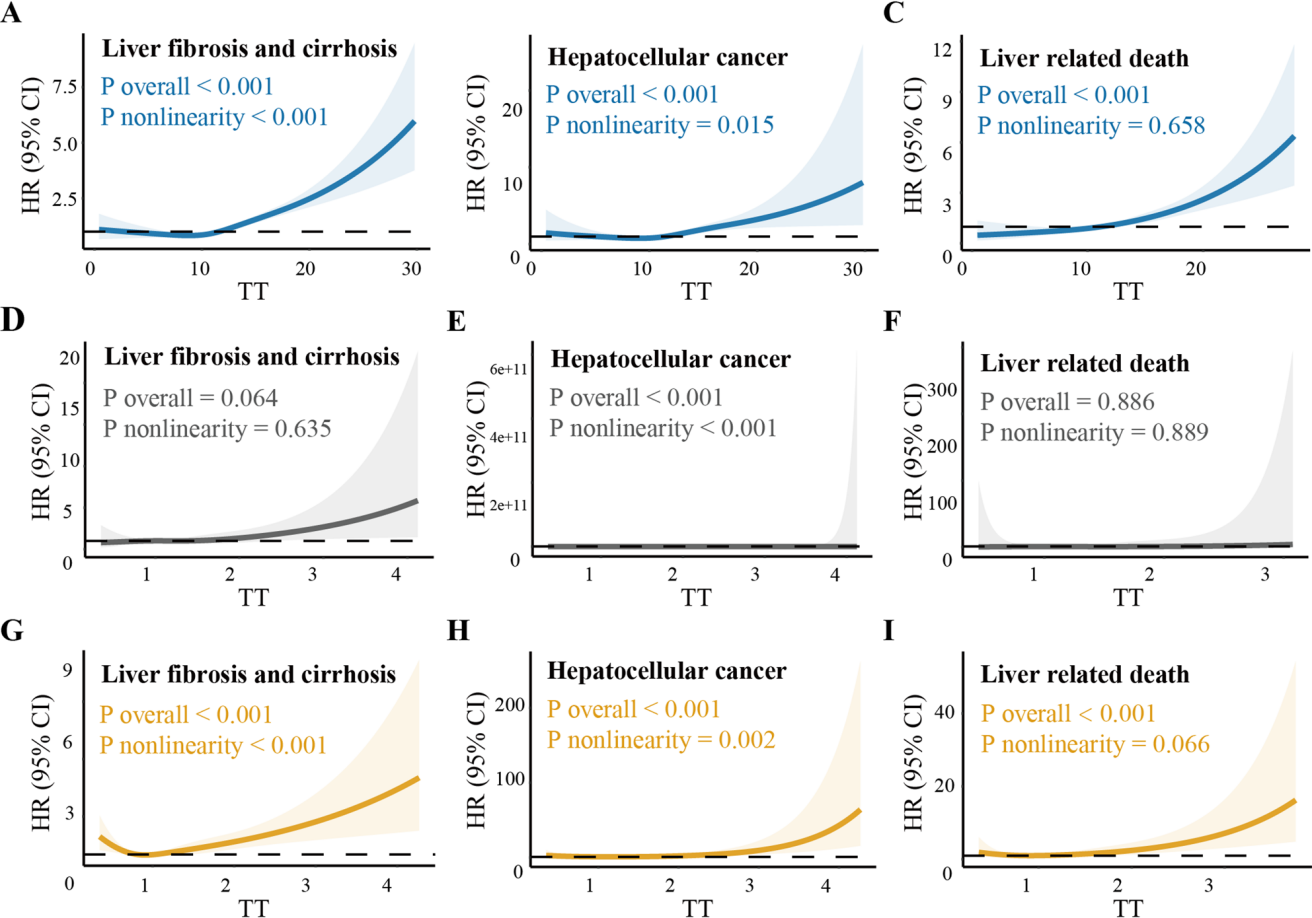
Restricted cubic spline analyses of the association between TT and chronic liver disease-related outcomes. **A**: for liver fibrosis and cirrhosis in males; **B**: for hepatocellular cancer in males; **C**: for liver related death in males; **D**: for liver fibrosis and cirrhosis in premenopausal females; **E**: for hepatocellular cancer in premenopausal females; **F**: for liver related death in premenopausal females; **G**: for liver fibrosis and cirrhosis in postmenopausal females; **H**: for hepatocellular cancer in postmenopausal females; **I**: for liver related death in postmenopausal females; Adjusted for age, race, qualifications, townsend deprivation index, smoking status, alcohol intake frequency, BMI status, physical activity, diet status, sleep status, and sedentary status, comorbidity (hypertension, diabetes, hyperlipidemia, ischemic heart disease, heart failure, stroke, renal failure) and medications (antihypertensive drugs, antidiabetic drugs, antihypertriglyceridemic drugs, antihypercholesterolemic drugs, steroids-related drug). TT: total testosterone; HR: hazard ratios; CI: confidence interval; BMI: body mass index

RCS analyses revealed significant associations between serum SHBG and TT levels and CLD–related outcomes, with distinct sex-specific patterns (Figs. 3 and 4). In men, higher SHBG and TT concentrations were strongly associated with increased risks of liver fibrosis and cirrhosis, and HCC (both *P* < 0.001), with a significant nonlinear trend observed for SHBG with HCC (P for nonlinearity = 0.030) and for TT with both outcomes (P for nonlinearity < 0.001 and 0.015, respectively), while the nonlinear trend for SHBG with liver fibrosis and cirrhosis was borderline (P for nonlinearity = 0.052). Both hormones also showed linear associations with liver-related death (*P* < 0.001). In premenopausal women, SHBG and TT exhibited weak or nonsignificant associations with CLD outcomes. In postmenopausal women, SHBG levels were positively and approximately linearly associated with all three outcomes (all *P* < 0.001; P for nonlinearity > 0.4), whereas TT levels were positively and nonlinearly associated with risks of liver fibrosis and cirrhosis (P for nonlinearity < 0.001) and HCC (P for nonlinearity = 0.002), but linearly associated with liver-related death (P for nonlinearity = 0.066). Overall, elevated SHBG and TT concentrations were consistently associated with higher risks of CLD-related outcomes, particularly among men and postmenopausal women.

### Cox analyses of associations between sex hormones and CLD-related outcomes across sex

**Fig. 5:**
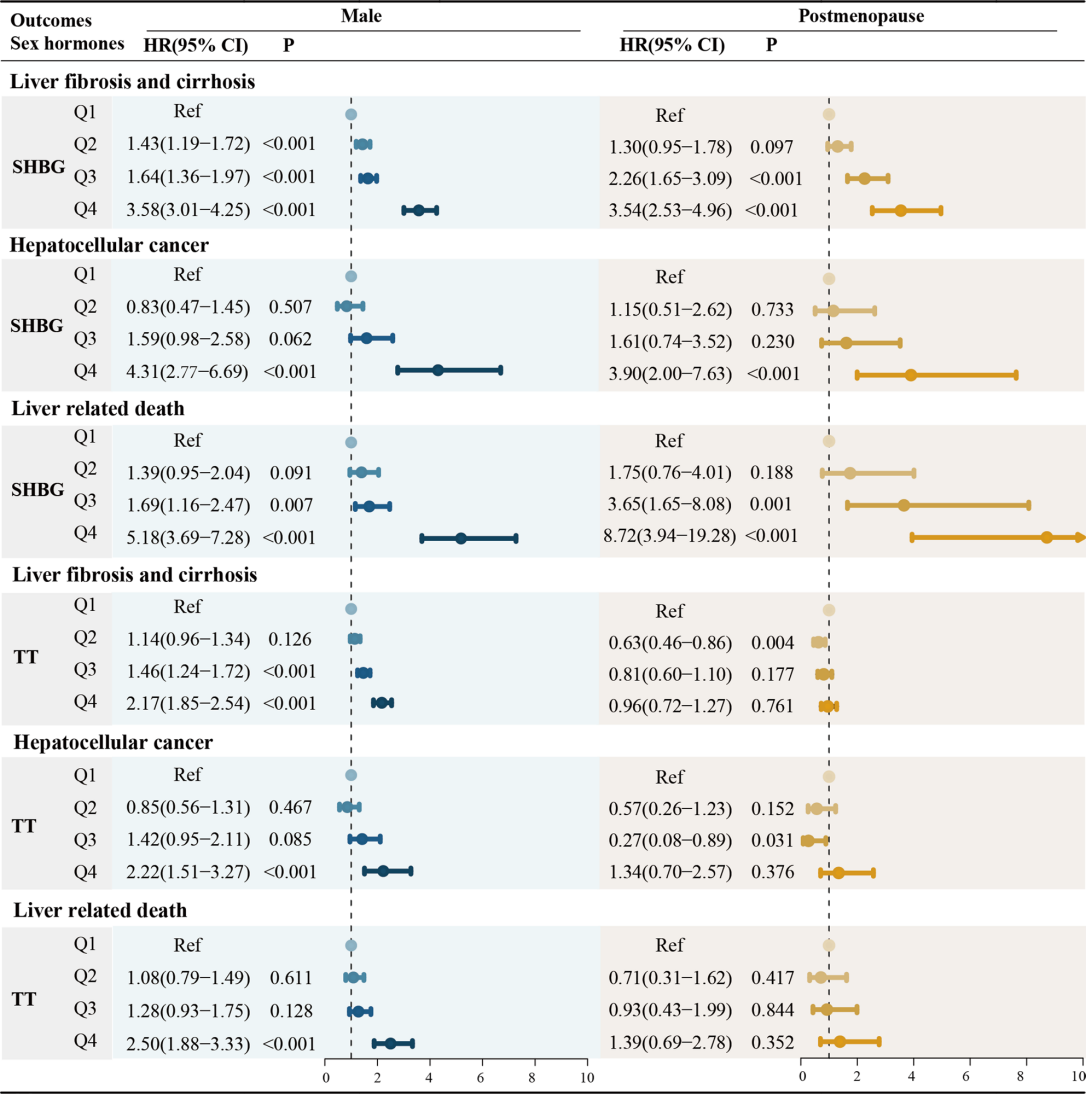
Cox proportional hazards analyses of associations between sex hormones and chronic liver disease-related outcomes. Adjusted for age, race, qualifications, townsend deprivation index, smoking status, alcohol intake frequency, alcohol intake per day, BMI status, physical activity, diet status, sleep status, and sedentary status, ALT, AST, ALB, TBIL, Uric, Crea, FIB4, comorbidity (hypertension, diabetes, hyperlipidemia, ischemic heart disease, heart failure, stroke, renal failure) and medications (antihypertensive drugs, antidiabetic drugs, antihypertriglyceridemic drugs, antihypercholesterolemic drugs, steroids-related drug), baseline liver diseases status (MASLD, MetALD, ALD, viral hepatitis). HR: hazard ratios; CI: confidence interval; SHBG: sex hormone-binding globulin; TT: total testosterone; Q1: quartile 1; Q2: quartile 2; Q3: quartile 3; Q4: quartile 4; Ref: reference; BMI: body mass index

Multivariable Cox proportional hazards models, adjusted for age, race, educational qualifications, Townsend Deprivation Index, smoking, alcohol intake frequency, alcohol intake per day, BMI category, physical activity, diet, sleep, sedentary status, ALT, AST, ALB, TBIL, Uric, Crea, comorbidities, and medication use, baseline liver diseases (MASLD, MetALD, ALD, viral hepatitis), showed strong positive associations of SHBG and TT with CLD–related outcomes (Fig. 5). Among men, higher SHBG quartiles were associated with increased risks of liver fibrosis and cirrhosis (HRs = 1.43 [95% CI: 1.19–1.72] for Q2; 1.64 [1.36–1.97] for Q3; 3.58 [3.01–4.25] for Q4; *P* < 0.001), HCC (HRs = 1.59 [0.98–2.58] for Q3, *P* = 0.062; 4.31 [2.77–6.69] for Q4, *P* < 0.001), and liver-related death (HRs = 1.69 [1.16–2.47] for Q3, *P* = 0.007; 5.18 [3.69–7.28] for Q4; *P* < 0.001). TT showed a similar dose–response pattern for fibrosis and cirrhosis (HRs = 1.46 [1.24–1.72] for Q3; 2.17 [1.85–2.54] for Q4; *P* < 0.001), HCC (HR = 2.22 [1.51–3.27] for Q4; *P* < 0.001), and liver-related death (HR = 2.50 [1.88–3.33]; *P* < 0.001). In postmenopausal women, higher SHBG levels were also associated with greater risks of fibrosis and cirrhosis, HCC, and liver-related death. TT in postmenopausal women was not significantly associated with CLD-related outcomes. In premenopausal women, no significant associations were observed (Supplementary Fig. 1) due to the limited number of events. Given the long follow-up period and potential time-varying effects, the proportional hazards assumption for Cox models was tested (Supplemental Table 7). The association between SHBG and liver fibrosis and cirrhosis violated this assumption in men (*P* < 0.001) and postmenopausal women (*P* = 0.001); therefore, follow-up time was stratified into two intervals (≤ 10 years and > 10 years), and the results were broadly consistent with those from the primary analyses (Supplemental Table 8). Furthermore, to account for the competing risk of death from other causes, Fine-Gray competing risks regression was applied to analyses of liver-related death, yielding results broadly consistent with those from the primary analyses (Supplemental Table 9). When SHBG and TT were mutually adjusted in the same model, SHBG remained significantly associated with CLD-related outcomes in both men and postmenopausal women, while the association of TT was attenuated and no longer statistically significant, potentially reflecting the high correlation between the two hormones (Supplemental Table 10). Subgroup analyses by age (< 65 vs. ≥ 65 years) and BMI (normal vs. overweight/obese), baseline liver diseases status (with vs. without liver disease at baseline) yielded results consistent with those of the main analyses (Supplementary Tables 11–16).

## Discussion

In this large population-based cohort study from the UKB, we comprehensively examined the associations of serum TT and SHBG with CLD-related outcomes, including liver fibrosis and cirrhosis, HCC, and liver-related death, in men, premenopausal women, and postmenopausal women. Over a median follow-up of approximately 13.6 years, we observed clear sex-specific patterns. Men exhibited substantially higher risks of all CLD-related outcomes compared with both premenopausal and postmenopausal women. Elevated SHBG and TT concentrations were associated with increased risks of liver fibrosis and cirrhosis, HCC, and liver-related death, particularly among men, weaker in postmenopausal women, whereas no significant associations were found in premenopausal women. These findings indicate that androgen-related signaling may associate with the pathogenesis and prognosis of CLD, with distinct effects according to sex and menopausal status.

Previous studies exploring the influence of androgens on liver disease have been relatively limited and yielded conflicting results, highlighting the complexity of sex hormone effects in hepatic pathophysiology. Some investigations have suggested that low testosterone predicts worse outcomes in men with advanced cirrhosis, reflecting secondary hypogonadism as a marker of severe hepatic dysfunction [21–23]. In contrast, others have reported that higher testosterone levels are associated with increased risk of HCV-related hepatic fibrosis [24] and HCC [25, 27], implying a potential pathogenic role of androgen excess. A Mendelian randomization (MR) study examining genetically predicted circulating TT levels and liver cancer risk found a non-significant positive association in males [34]. Our findings reinforce previous reports by showing a consistent association between elevated TT levels and a full spectrum of CLD outcomes, including liver fibrosis, cirrhosis, HCC, and liver-related death in men. This highlights the consistent role of testosterone in CLD progression in men. The lack of research in women, particularly across the menopausal transition, has further constrained understanding of these relationships. MR analyses have suggested no causal association between TT levels and HCC in females [35]. We found that, in postmenopausal women, elevated testosterone levels were still associated with increased risks of liver disease outcomes, although this association was weaker compared to men. Previous studies have also indicated that the relationship between TT levels and HCC in postmenopausal women is weaker than the corresponding association observed in men [27]. In contrast, in premenopausal women, where testosterone levels are naturally lower, this risk association disappeared, possibly due to the inherently lower androgen levels and the relatively small number of outcome events observed in this group.

Recent mechanistic studies have provided important insights into how androgens influence liver fibrosis and hepatocarcinogenesis. Previous data have shown that inflammation could be regulated by sex hormones by modulating the expression of inflammatory signaling pathways [36, 37]. It has been found that androgens exacerbate hepatic fibrosis by promoting activation of the NLRP3 inflammasome [28], leading to excessive inflammatory responses and increased secretion of pro-inflammatory cytokines. Furthermore, Bizzaro et al. demonstrated a sexual dimorphism in hepatic inflammation and repair in a murine model of acute liver injury, showing that androgen receptor (AR) signaling promotes the recruitment of pro-inflammatory immature monocytes to the male liver, thereby sustaining inflammatory responses [38]. At the carcinogenic stage, increased androgen levels, overexpression of AR, and enhanced activity of androgen response elements have been observed in HCC [29, 39, 40]. Mechanistically, androgen facilitates hepatitis B virus replication by binding to androgen-responsive elements within the viral enhancer I region, and HBV X protein further augments AR transactivation via c-Src and glycogen synthase kinase-3 signaling [29, 40, 41]. AR also contributes to hepatocarcinogenesis through upregulation of miR-216a and suppression of the tumor suppressor TSLC1, as well as forming a positive feedback loop with cell cycle-related kinase and β-catenin to sustain Wnt/β-catenin pathway activation [30, 31]. Beyond hormonal signaling, redox imbalance also contributes to chronic liver disease progression by promoting hepatic stellate cell activation, DNA damage, and pro-inflammatory cytokine release. Circulating biomarkers such as bilirubin and creatinine have been reported to be associated with cancer risk [42–44], suggesting that systemic metabolic and redox status may modulate oncogenic processes across multiple organ systems. The potential interplay between androgen signaling and hepatic oxidative stress represents an important direction for future mechanistic investigation. These molecular mechanisms collectively indicate that androgen signaling amplifies inflammatory and proliferative responses in the liver, accelerating fibrosis and predisposing to malignant transformation.

SHBG is a glycoprotein primarily produced in the liver and is reported to have associations with liver dysfunction. Elevated SHBG levels are commonly observed in individuals with late-stage chronic liver disease [45], and higher concentrations of SHBG have been linked to liver cirrhosis. Recent data indicate that SHBG accumulates in fibrotic regions of the liver and its expression is inversely correlated with fibrosis severity in humans [46]. Furthermore, SHBG has been associated with an increased risk of HCC [27], suggesting its potential role as a marker of disease severity. The results of a Mendelian randomization study suggested that SHBG may not be causally linked to liver cancer risk in either males or females, but rather may act as a consequence of liver injury [47]. Our study found that elevated SHBG levels were associated with increased liver disease risk, including liver fibrosis and cirrhosis, HCC and liver-related death. Mechanistic studies using SHBG-transgenic mice revealed that SHBG impeded liver fibrosis progression by downregulating TGF-β signaling [48], and inhibited hepatocellular carcinoma via its action on myeloid-epithelial reproductive tyrosine kinase [49]. These contrasting findings highlight the need for further research to explore the causal relationship and underlying mechanisms of SHBG in liver disease progression. Importantly, when TT and SHBG were mutually adjusted in the same model, SHBG retained its significant association with CLD-related outcomes, whereas the association of TT was attenuated. This likely reflects the high correlation between the two hormones, as SHBG is the primary binding protein for testosterone, making it statistically challenging to fully disentangle their independent contributions. The robust associations of TT observed in the primary analyses, supported by mechanistic evidence, continue to suggest a role for androgen signaling in liver disease progression. Future studies incorporating free or bioavailable testosterone may help clarify their respective contributions.

This study also had some limitations. UKB measured three sex hormones, including TT, SHBG and oestradiol. However, oestradiol levels were not analyzed in this study because the majority of measurements were below the detection limit in men and postmenopausal women. Other sex hormones such as free testosterone, progesterone, and dihydrotestosterone were not measured in the UKB dataset. The absence of free or bioavailable testosterone measurements limits our ability to fully disentangle the independent effects of TT and SHBG, given their biological interdependence. Sex hormones exhibit substantial diurnal variation, with testosterone levels peaking in the early morning and declining throughout the day. In men, this variation can be clinically significant, and non-standardized sampling times may lead to underestimation of true TT levels. In premenopausal women, additional variability arises from hormonal fluctuations across the menstrual cycle. As blood samples in the UK Biobank were not consistently collected during the morning under fasting conditions, measurement inconsistency may have introduced non-differential exposure misclassification, potentially attenuating the observed associations. Moreover, the use of a single time-point measurement cannot fully capture long-term hormonal exposure trajectories, which may be more relevant to the development of chronic liver disease outcomes. Future studies with repeated hormone measurements would provide a more accurate characterization of long-term androgen exposure. Additionally, the low incidence and heterogeneity of CLD-related outcomes, particularly among premenopausal women, may have affected statistical power. The UKB cohort predominantly consists of individuals of European ancestry, which may limit the generalizability of the results across different race. Future studies with more comprehensive hormone assessments and more diverse populations are needed for further validation.

## Conclusion

In this large population-based cohort study, we found that elevated levels of TT and SHBG were associated with increased risks of CLD-related outcomes, including liver fibrosis and cirrhosis, HCC, and liver-related death, particularly in men, and weaker in postmenopausal women, while no significant associations were observed in premenopausal women. These findings underscore the importance of androgen-related signaling in CLD progression, with distinct effects observed based on sex and menopausal status. Future studies should focus on elucidating the causal relationships and underlying mechanisms of sex hormones in liver disease progression.

## Supplementary Information

Supplementary material 1. Supplemental Table 1. Baseline characteristics of male population based on SHBG quartiles. Supplemental Table 2. Baseline characteristics of male population based on TT quartiles. Supplemental Table 3. Baseline characteristics of postmenopausal female population based on SHBG quartiles. Supplemental Table 4. Baseline characteristics of postmenopausal female population based on TT quartiles.Supplemental Table 5. Baseline characteristics of premenopausal female population based on SHBG quartiles. Supplemental Table 6. Baseline characteristics of premenopausal female population based on TT quartiles. Supplemental table 7. Testing the proportional hazards assumption for Cox regression models. Supplemental Table 8. Time-stratified sensitivity analyses for associations between SHBG and liver fibrosis and cirrhosis. Supplemental Table 9. Fine-Gray competing risks analyses for liver-related death. Supplemental Table 10. Sensitivity analyses with mutual adjustment of SHBG and TT in Cox regression models for CLD-related outcomes. Supplemental Table 11. Cox analysis of sex hormone and chronic liver disease based on age subgroup in male population. Supplemental Table 12. Cox analysis of sex hormone and chronic liver disease based on BMI subgroup in male population. Supplemental Table 13. Cox analysis of sex hormone and chronic liver disease based on baseline liver diseases status in male population. Supplemental Table 14. Cox analysis of sex hormone and chronic liver disease based on age subgroup in postmenopausal female population. Supplemental Table 15. Cox analysis of sex hormone and chronic liver disease based on BMI subgroup in postmenopausal female population. Supplemental Table 16. Cox analysis of sex hormone and chronic liver disease based on baseline liver diseases status in postmenopausal female population. Supplemental Figure 1. Cox analyses of associations between sex hormones and chronic liver disease-related outcomes in premenopausal females.

## Data Availability

Researchers can apply for access to the UK Biobank database by submitting an application, which must include a summary of the research plan, the required data fields, any new variables to be generated, and payment to cover the associated costs. For more details, researchers can visit the UK Biobank’s website: https://www.ukbiobank.ac.uk/enable-your-research/apply-for-access. This study used data from the UK Biobank (application number 89483).

## Abbreviations

ALD: Alcohol-related liver disease
AR: Androgen receptor
BMI: Body mass index
CIs: Confidence intervals
CLD: Chronic liver disease
HCC: Hepatocellular carcinoma
HRs: Hazard ratios
ICD-10: International Classification of Diseases, 10th Revision
MASLD: Metabolic dysfunction associated steatotic liver disease
MR: Mendelian randomization
RCS: Restricted cubic spline
SHBG: Sex hormone-binding globulin
TDI: Townsend Deprivation Index
TT: Total testosterone
UKB: UK Biobank
