# Supplementary material for: Association of testosterone and sex hormone–binding globulin with chronic liver disease outcomes across sex and menopausal status
Source: Biol Sex Differ. 2026 May 21;17:146. doi: 10.1186/s13293-026-00923-8 (PMC13474746; doi:10.1186/s13293-026-00923-8)
Supplement: Supplementary file 1 [file 13293_2026_923_MOESM1_ESM.docx]

Supplemental Table 1 Baseline characteristics of male population based on SHBG quartiles.

|  | Q1^#^ | Q2 | Q3 | Q4 |  |
| --- | --- | --- | --- | --- | --- |
| N | 39056 | 39061 | 39060 | 38820 |  |
| **‌Sociodemographic factors** |  |  |  |  |  |
| Age (years) | 53.59(8.11) | 56.19(8.05) | 57.90(7.79) | 59.67(7.29) | <0.001 |
| Race (white, %) | 35991(92.2) | 37003(94.7) | 37417(95.8) | 37515(96.6) | <0.001 |
| Qualifications (%) |  |  |  |  | <0.001 |
| College or University | 13760(35.2) | 13670(35.0) | 13627(34.9) | 13017(33.5) |  |
| A levels/AS levels | 4477(11.5) | 4250(10.9) | 3877(9.9) | 3670(9.5) |  |
| O levels/GCSEs | 7843(20.1) | 7539(19.3) | 7270(18.6) | 6897(17.8) |  |
| CSEs | 2732(7.0) | 2286(5.9) | 1908(4.9) | 1625(4.2) |  |
| NVQ or HND or HNC | 3437(8.8) | 3577(9.2) | 3607(9.2) | 3689(9.5) |  |
| Other professional qualifications | 1565(4.0) | 1722(4.4) | 1811(4.6) | 1910(4.9) |  |
| None of the above | 5242(13.4) | 6017(15.4) | 6960(17.8) | 8012(20.6) |  |
| TDI (%) |  |  |  |  | <0.001 |
| Tertile 1 | 12583(32.2) | 13528(34.6) | 13659(35.0) | 13229(34.1) |  |
| Tertile 2 | 12866(32.9) | 13076(33.5) | 13168(33.7) | 12810(33.0) |  |
| Tertile 3 | 13607(34.8) | 12457(31.9) | 12233(31.3) | 12781(32.9) |  |
| **Lifestyle factors** |  |  |  |  |  |
| Smoking status (%) |  |  |  |  | <0.001 |
| Never | 19757(50.6) | 19191(49.1) | 18668(47.8) | 18114(46.7) |  |
| Ever | 15357(39.3) | 15588(39.9) | 15602(39.9) | 14799(38.1) |  |
| Current | 3942(10.1) | 4282(11.0) | 4790(12.3) | 5907(15.2) |  |
| Alcohol intake per day (g) | 23.42(22.62) | 23.10(22.08) | 22.76(21.79) | 21.13(22.36) | <0.001 |
| Drinking status (%) |  |  |  |  | <0.001 |
| Never or special occasions | 5786(14.8) | 5167(13.2) | 5067(13.0) | 6197(16.0) |  |
| Monthly to weekly | 23277(59.6) | 23040(59.0) | 22877(58.6) | 21948(56.5) |  |
| Never or special occasions | 9993(25.6) | 10854(27.8) | 11116(28.5) | 10675(27.5) |  |
| Physical status (%) |  |  |  |  | <0.001 |
| Unhealthy | 23598(60.4) | 22517(57.6) | 21404(54.8) | 20137(51.9) |  |
| Healthy | 8777(22.5) | 9859(25.2) | 10934(28.0) | 11742(30.2) |  |
| Miss | 6681(17.1) | 6685(17.1) | 6722(17.2) | 6941(17.9) |  |
| Diet status (%) |  |  |  |  | <0.001 |
| Unhealthy | 33335(85.4) | 32598(83.5) | 31929(81.7) | 30814(79.4) |  |
| Healthy | 5257(13.5) | 6059(15.5) | 6728(17.2) | 7578(19.5) |  |
| Miss | 464(1.2) | 404(1.0) | 403(1.0) | 428(1.1) |  |
| Sleep status (%) |  |  |  |  | <0.001 |
| Unhealthy | 4679(12.0) | 4613(11.8) | 4711(12.1) | 4985(12.8) |  |
| Healthy | 34235(87.7) | 34339(87.9) | 34231(87.6) | 33694(86.8) |  |
| Miss | 142(0.4) | 109(0.3) | 118(0.3) | 141(0.4) |  |
| Sedentary status (%) |  |  |  |  | <0.001 |
| Unhealthy | 31836(81.5) | 31553(80.8) | 31206(79.9) | 30570(78.7) |  |
| Healthy | 6972(17.9) | 7298(18.7) | 7647(19.6) | 7992(20.6) |  |
| Miss | 248(0.6) | 210(0.5) | 207(0.5) | 258(0.7) |  |
| BMI status (%) |  |  |  |  | <0.001 |
| Underweight (<18.5) | 5(0.0) | 19(0.0) | 55(0.1) | 256(0.7) |  |
| Normal (18.5-24.99) | 4577(11.7) | 7691(19.7) | 10809(27.7) | 15757(40.6) |  |
| Overweight (25.0-29.99) | 19867(50.9) | 20636(52.8) | 19938(51.0) | 17271(44.5) |  |
| Obesity (>=30.0) | 14607(37.4) | 10715(27.4) | 8258(21.1) | 5536(14.3) |  |
| **Comorbidity and medications** |  |  |  |  |  |
| T2DM (%) | 4194(10.7) | 2707(6.9) | 2240(5.7) | 1940(5.0) | <0.001 |
| Hypertension (%) | 32626(83.5) | 32018(82.0) | 31616(80.9) | 30672(79.0) | <0.001 |
| Hyperlipidemia (%) | 36242(92.8) | 34899(89.3) | 33958(86.9) | 32311(83.2) | <0.001 |
| Ischemic heart disease (%) | 2963(7.6) | 3172(8.1) | 3377(8.6) | 3082(7.9) | <0.001 |
| Heart failure (%) | 298(0.8) | 297(0.8) | 318(0.8) | 379(1.0) | 0.002 |
| Stroke (%) | 650(1.7) | 685(1.8) | 740(1.9) | 915(2.4) | <0.001 |
| Renal failure (%) | 680(1.7) | 637(1.6) | 607(1.6) | 558(1.4) | 0.007 |
| Antidiabetic drugs (%) | 2976(7.6) | 1911(4.9) | 1605(4.1) | 1462(3.8) | <0.001 |
| Antihypertensive drugs (%) | 12052(30.9) | 12115(31.0) | 12168(31.2) | 11540(29.7) | <0.001 |
| Antihypertriglyceridemic drugs (%) | 393(1.0) | 301(0.8) | 278(0.7) | 288(0.7) | <0.001 |
| Antihypercholesterolemic drugs (%) | 11509(29.5) | 10833(27.7) | 10677(27.3) | 9789(25.2) | <0.001 |
| Steroids-related drug (%) | 1901(4.9) | 1814(4.6) | 1687(4.3) | 1716(4.4) | 0.001 |
| **Liver diseases and liver status** |  |  |  |  |  |
| MASLD (%) | 19539(50.0) | 15443(39.5) | 12452(31.9) | 8901(22.9) | <0.001 |
| MetALD (%) | 6918(17.7) | 5767(14.8) | 4532(11.6) | 3009(7.8) | <0.001 |
| ALD (%) | 2335(6.0) | 1768(4.5) | 1586(4.1) | 1117(2.9) | <0.001 |
| Viral hepatitis (%) | 29(0.1) | 28(0.1) | 30(0.1) | 82(0.2) | <0.001 |
| ALT (U/L) | 31.52(15.96) | 27.77(14.26) | 25.79(13.60) | 24.42(15.24) | <0.001 |
| AST (U/L) | 28.64(9.98) | 27.84(9.15) | 27.75(10.50) | 28.53(12.29) | <0.001 |
| ALB (g/L) | 45.95(2.56) | 45.64(2.55) | 45.45(2.57) | 45.24(2.64) | <0.001 |
| TBIL (umol/L) | 9.91(4.52) | 10.27(4.80) | 10.42(4.86) | 10.67(5.12) | <0.001 |
| Uric (umol/L) | 377.58(72.81) | 361.57(69.85) | 349.95(67.81) | 333.17(67.85) | <0.001 |
| Crea (umol/L) | 82.61(19.50) | 82.48(18.58) | 81.80(18.08) | 80.37(17.14) | <0.001 |
| FIB4 | 1.24(0.95) | 1.37(0.62) | 1.50(4.77) | 1.63(0.85) | <0.001 |
| **Sex hormones** |  |  |  |  |  |
| SHBG (nmol/L) | 21.82(4.53) | 32.49(2.57) | 42.11(3.18) | 61.21(12.22) | <0.001 |
| TT (nmol/L) | 9.44(2.62) | 11.10(2.79) | 12.47(3.00) | 14.69(3.61) | <0.001 |
| E2 (pmol/L)* | 223.38(78.49) | 223.09(78.85) | 220.18(62.60) | 221.22(85.88) | 0.298 |
| **Follow-up outcomes** |  |  |  |  |  |
| Liver fibrosis and cirrhosis (%) | 217(0.6) | 260(0.7) | 271(0.7) | 567(1.5) | <0.001 |
| Hepatocellular cancer (%) | 29(0.1) | 22(0.1) | 42(0.1) | 119(0.3) | <0.001 |
| Liver related death (%) | 49(0.1) | 59(0.2) | 67(0.2) | 218(0.6) | <0.001 |

TDI: townsend deprivation index; T2DM: type 2 diabetes mellitus; MASLD: Metabolic dysfunction-associated steatotic liver disease; MetALD: Metabolic dysfunction-associated steatotic liver disease with increased alcohol intake; ALD: alcohol-related liver disease; ALT: alanine aminotransferase; AST: aspartate aminotransferase; ALB: albumin; TBIL: total bilirubin; Uric: uric acid; Crea: creatinine; FIB4: fibrosis-4 index; SHBG: sex hormone binding globulin; TT: total testosterone; E2: oestradiol.

Continuous variables (age, SHBG, TT and E2) are presented as means (standard deviations), and categorical variables are expressed as N (percentages). The t-test was employed to compare differences in continuous variables, while chi-square tests were used for categorical variables.

^#^Participants were categorized into quartiles based on sex hormone binding globulin levels.

*Among them, E2 was measured in 13,144 men.

Supplemental Table 2 Baseline characteristics of male population based on TT quartiles.

|  | Q1^#^ | Q2 | Q3 | Q4 |  |
| --- | --- | --- | --- | --- | --- |
| N | 39056 | 39068 | 39063 | 38810 |  |
| **‌Sociodemographic factors** |  |  |  |  |  |
| Age (years) | 57.61(7.90) | 56.99(8.09) | 56.69(8.16) | 56.03(8.31) | <0.001 |
| Race (white, %) | 36980(94.7) | 37056(94.9) | 37059(94.9) | 36831(94.9) | 0.525 |
| Qualifications (%) |  |  |  |  | <0.001 |
| College or University | 12471(31.9) | 13588(34.8) | 14030(35.9) | 13985(36.0) |  |
| A levels/AS levels | 4031(10.3) | 4041(10.3) | 4116(10.5) | 4086(10.5) |  |
| O levels/GCSEs | 7480(19.2) | 7441(19.0) | 7274(18.6) | 7354(18.9) |  |
| CSEs | 2005(5.1) | 2110(5.4) | 2099(5.4) | 2337(6.0) |  |
| NVQ or HND or HNC | 3806(9.7) | 3594(9.2) | 3559(9.1) | 3351(8.6) |  |
| Other professional qualifications | 1871(4.8) | 1795(4.6) | 1722(4.4) | 1620(4.2) |  |
| None of the above | 7392(18.9) | 6499(16.6) | 6263(16.0) | 6077(15.7) |  |
| TDI (%) |  |  |  |  | <0.001 |
| Tertile 1 | 13137(33.6) | 13670(35.0) | 13451(34.4) | 12741(32.8) |  |
| Tertile 2 | 13049(33.4) | 13200(33.8) | 13086(33.5) | 12585(32.4) |  |
| Tertile 3 | 12870(33.0) | 12198(31.2) | 12526(32.1) | 13484(34.7) |  |
| **Lifestyle factors** |  |  |  |  |  |
| Smoking status (%) |  |  |  |  | <0.001 |
| Never | 17701(45.3) | 19066(48.8) | 19398(49.7) | 19565(50.4) |  |
| Ever | 17482(44.8) | 15694(40.2) | 14905(38.2) | 13265(34.2) |  |
| Current | 3873(9.9) | 4308(11.0) | 4760(12.2) | 5980(15.4) |  |
| Alcohol intake per day (g) | 22.45(22.17) | 22.85(22.14) | 22.75(21.86) | 22.37(22.74) | 0.005 |
| Drinking status (%) |  |  |  |  | <0.001 |
| Never or special occasions | 6247(16.0) | 5283(13.5) | 5088(13.0) | 5599(14.4) |  |
| Monthly to weekly | 22359(57.2) | 22949(58.7) | 22916(58.7) | 22918(59.1) |  |
| Never or special occasions | 10450(26.8) | 10836(27.7) | 11059(28.3) | 10293(26.5) |  |
| Physical status (%) |  |  |  |  | <0.001 |
| Unhealthy | 22788(58.3) | 22186(56.8) | 21735(55.6) | 20947(54.0) |  |
| Healthy | 9072(23.2) | 10181(26.1) | 10770(27.6) | 11289(29.1) |  |
| Miss | 7196(18.4) | 6701(17.2) | 6558(16.8) | 6574(16.9) |  |
| Diet status (%) |  |  |  |  | <0.001 |
| Unhealthy | 32505(83.2) | 32506(83.2) | 32057(82.1) | 31608(81.4) |  |
| Healthy | 6092(15.6) | 6199(15.9) | 6586(16.9) | 6745(17.4) |  |
| Miss | 459(1.2) | 363(0.9) | 420(1.1) | 457(1.2) |  |
| Sleep status (%) |  |  |  |  | <0.001 |
| Unhealthy | 5529(14.2) | 4667(11.9) | 4330(11.1) | 4462(11.5) |  |
| Healthy | 33367(85.4) | 34294(87.8) | 34624(88.6) | 34214(88.2) |  |
| Miss | 160(0.4) | 107(0.3) | 109(0.3) | 134(0.3) |  |
| Sedentary status (%) |  |  |  |  | <0.001 |
| Unhealthy | 32441(83.1) | 31518(80.7) | 30954(79.2) | 30252(77.9) |  |
| Healthy | 6369(16.3) | 7347(18.8) | 7862(20.1) | 8331(21.5) |  |
| Miss | 246(0.6) | 203(0.5) | 247(0.6) | 227(0.6) |  |
| BMI status (%) |  |  |  |  | <0.001 |
| Underweight (<18.5) | 41(0.1) | 47(0.1) | 73(0.2) | 174(0.4) |  |
| Normal (18.5-24.99) | 5166(13.2) | 8033(20.6) | 10927(28.0) | 14708(37.9) |  |
| Overweight (25.0-29.99) | 18278(46.8) | 20487(52.4) | 20225(51.8) | 18722(48.2) |  |
| Obesity (>=30.0) | 15571(39.9) | 10501(26.9) | 7838(20.1) | 5206(13.4) |  |
| **Comorbidity and medications** |  |  |  |  |  |
| T2DM (%) | 4876(12.5) | 2737(7.0) | 2013(5.2) | 1455(3.7) | <0.001 |
| Hypertension (%) | 33919(86.8) | 32515(83.2) | 31269(80.0) | 29229(75.3) | <0.001 |
| Hyperlipidemia (%) | 36224(92.7) | 35057(89.7) | 33879(86.7) | 32250(83.1) | <0.001 |
| Ischemic heart disease (%) | 4230(10.8) | 3307(8.5) | 2834(7.3) | 2223(5.7) | <0.001 |
| Heart failure (%) | 430(1.1) | 310(0.8) | 268(0.7) | 284(0.7) | <0.001 |
| Stroke (%) | 917(2.3) | 774(2.0) | 688(1.8) | 611(1.6) | <0.001 |
| Renal failure (%) | 843(2.2) | 622(1.6) | 585(1.5) | 432(1.1) | <0.001 |
| Antidiabetic drugs (%) | 3510(9.0) | 1960(5.0) | 1420(3.6) | 1064(2.7) | <0.001 |
| Antihypertensive drugs (%) | 15509(39.7) | 12454(31.9) | 10935(28.0) | 8977(23.1) | <0.001 |
| Antihypertriglyceridemic drugs (%) | 460(1.2) | 309(0.8) | 281(0.7) | 210(0.5) | <0.001 |
| Antihypercholesterolemic drugs (%) | 14744(37.8) | 11273(28.9) | 9490(24.3) | 7301(18.8) | <0.001 |
| Steroids-related drug (%) | 2174(5.6) | 1821(4.7) | 1622(4.2) | 1501(3.9) | <0.001 |
| **Liver diseases and liver status** |  |  |  |  |  |
| MASLD (%) | 19718(50.5) | 15485(39.6) | 12393(31.7) | 8739(22.5) | <0.001 |
| MetALD (%) | 6438(16.5) | 5629(14.4) | 4763(12.2) | 3396(8.8) | <0.001 |
| ALD (%) | 2137(5.5) | 1847(4.7) | 1529(3.9) | 1293(3.3) | <0.001 |
| Viral hepatitis (%) | 32(0.1) | 33(0.1) | 21(0.1) | 83(0.2) | <0.001 |
| ALT (U/L) | 29.62(15.35) | 27.84(15.01) | 26.63(14.50) | 25.43(14.93) | <0.001 |
| AST (U/L) | 28.59(10.43) | 28.06(9.98) | 27.97(10.12) | 28.15(11.58) | <0.001 |
| ALB (g/L) | 45.49(2.62) | 45.59(2.57) | 45.59(2.58) | 45.60(2.61) | <0.001 |
| TBIL (umol/L) | 9.58(4.43) | 10.13(4.65) | 10.51(4.85) | 11.04(5.28) | <0.001 |
| Uric (umol/L) | 372.33(75.13) | 360.46(70.61) | 351.33(68.54) | 338.18(66.95) | <0.001 |
| Crea (umol/L) | 82.67(22.43) | 82.11(16.06) | 81.69(16.23) | 80.78(17.99) | <0.001 |
| FIB4 | 1.40(0.94) | 1.41(0.81) | 1.43(0.64) | 1.49(4.80) | <0.001 |
| **Sex hormones** |  |  |  |  |  |
| SHBG (nmol/L) | 28.67(11.08) | 35.30(11.80) | 41.55(13.34) | 52.06(16.99) | <0.001 |
| TT (nmol/L) | 7.74(1.39) | 10.52(0.62) | 12.76(0.71) | 16.71(2.39) | <0.001 |
| E2 (pmol/L)* | 225.78(91.40) | 220.32(70.64) | 221.38(71.54) | 221.28(78.03) | 0.075 |
| **Follow-up outcomes** |  |  |  |  |  |
| Liver fibrosis and cirrhosis (%) | 316(0.8) | 271(0.7) | 312(0.8) | 416(1.1) | <0.001 |
| Hepatocellular cancer (%) | 58(0.1) | 35(0.1) | 50(0.1) | 69(0.2) | 0.008 |
| Liver related death (%) | 90(0.2) | 74(0.2) | 78(0.2) | 151(0.4) | <0.001 |

TDI: townsend deprivation index; T2DM: type 2 diabetes mellitus; MASLD: Metabolic dysfunction-associated steatotic liver disease; MetALD: Metabolic dysfunction-associated steatotic liver disease with increased alcohol intake; ALD: alcohol-related liver disease; ALT: alanine aminotransferase; AST: aspartate aminotransferase; ALB: albumin; TBIL: total bilirubin; Uric: uric acid; Crea: creatinine; FIB4: fibrosis-4 index; SHBG: sex hormone binding globulin; TT: total testosterone; E2: oestradiol.

Continuous variables (age, alcohol intake per day, ALT, AST, ALB, TBIL, Uric, Crea, FIB4, SHBG, TT and E2) are presented as means (standard deviations), and categorical variables are expressed as N (percentages). The t-test was employed to compare differences in continuous variables, while chi-square tests were used for categorical variables.

^#^Participants were categorized into quartiles based on total testosterone levels.

*Among them, E2 was measured in 13,144 men.

Supplemental Table 3 Baseline characteristics of postmenopausal female population based on SHBG quartiles.

|  | Q1^#^ | Q2 | Q3 | Q4 |  |
| --- | --- | --- | --- | --- | --- |
| N | 21723 | 20833 | 19728 | 17353 |  |
| **‌Sociodemographic factors** |  |  |  |  |  |
| Age (years) | 60.11(5.31) | 60.55(5.33) | 60.45(5.39) | 60.56(5.48) | <0.001 |
| Race (white, %) | 20340(93.6) | 19948(95.8) | 19038(96.5) | 16849(97.1) | <0.001 |
| Qualifications (%) |  |  |  |  | <0.001 |
| College or University | 5699(26.2) | 5951(28.6) | 6092(30.9) | 5604(32.3) |  |
| A levels/AS levels | 2339(10.8) | 2211(10.6) | 2177(11.0) | 1970(11.4) |  |
| O levels/GCSEs | 5122(23.6) | 4956(23.8) | 4661(23.6) | 3944(22.7) |  |
| CSEs | 1029(4.7) | 842(4.0) | 716(3.6) | 644(3.7) |  |
| NVQ or HND or HNC | 1079(5.0) | 993(4.8) | 875(4.4) | 637(3.7) |  |
| Other professional qualifications | 1522(7.0) | 1378(6.6) | 1299(6.6) | 1203(6.9) |  |
| None of the above | 4933(22.7) | 4502(21.6) | 3908(19.8) | 3351(19.3) |  |
| TDI (%) |  |  |  |  | <0.001 |
| Tertile 1 | 7199(33.1) | 7267(34.9) | 7110(36.0) | 6119(35.3) |  |
| Tertile 2 | 7332(33.8) | 7314(35.1) | 6750(34.2) | 6028(34.7) |  |
| Tertile 3 | 7192(33.1) | 6252(30.0) | 5868(29.7) | 5206(30.0) |  |
| **Lifestyle factors** |  |  |  |  |  |
| Smoking status (%) |  |  |  |  | <0.001 |
| Never | 12525(57.7) | 12075(58.0) | 11650(59.1) | 10336(59.6) |  |
| Ever | 7713(35.5) | 7149(34.3) | 6430(32.6) | 5491(31.6) |  |
| Current | 1485(6.8) | 1609(7.7) | 1648(8.4) | 1526(8.8) |  |
| Alcohol intake per day (g) | 10.02(12.49) | 10.51(11.99) | 10.24(11.40) | 9.09(10.77) | <0.001 |
| Drinking status (%) |  |  |  |  | <0.001 |
| Never or special occasions | 6754(31.1) | 5304(25.5) | 4870(24.7) | 4910(28.3) |  |
| Monthly to weekly | 11077(51.0) | 11324(54.4) | 10903(55.3) | 9351(53.9) |  |
| Never or special occasions | 3892(17.9) | 4205(20.2) | 3955(20.0) | 3092(17.8) |  |
| Physical status (%) |  |  |  |  | <0.001 |
| Unhealthy | 11324(52.1) | 10530(50.5) | 9984(50.6) | 8508(49.0) |  |
| Healthy | 4178(19.2) | 4594(22.1) | 4740(24.0) | 4612(26.6) |  |
| Miss | 6221(28.6) | 5709(27.4) | 5004(25.4) | 4233(24.4) |  |
| Diet status (%) |  |  |  |  | <0.001 |
| Unhealthy | 16580(76.3) | 15361(73.7) | 14005(71.0) | 11602(66.9) |  |
| Healthy | 4943(22.8) | 5273(25.3) | 5565(28.2) | 5614(32.4) |  |
| Miss | 200(0.9) | 199(1.0) | 158(0.8) | 137(0.8) |  |
| Sleep status (%) |  |  |  |  | <0.001 |
| Unhealthy | 3351(15.4) | 2794(13.4) | 2528(12.8) | 2210(12.7) |  |
| Healthy | 18224(83.9) | 17927(86.1) | 17073(86.5) | 15039(86.7) |  |
| Miss | 148(0.7) | 112(0.5) | 127(0.6) | 104(0.6) |  |
| Sedentary status (%) |  |  |  |  | <0.001 |
| Unhealthy | 17561(80.8) | 16275(78.1) | 14987(76.0) | 12546(72.3) |  |
| Healthy | 3904(18.0) | 4320(20.7) | 4559(23.1) | 4642(26.8) |  |
| Miss | 258(1.2) | 238(1.1) | 182(0.9) | 165(1.0) |  |
| BMI status (%) |  |  |  |  | <0.001 |
| Underweight (<18.5) | 6(0.0) | 44(0.2) | 121(0.6) | 376(2.2) |  |
| Normal (18.5-24.99) | 3047(14.0) | 6324(30.4) | 9137(46.3) | 10572(60.9) |  |
| Overweight (25.0-29.99) | 8718(40.1) | 9202(44.2) | 7673(38.9) | 5175(29.8) |  |
| Obesity (>=30.0) | 9952(45.8) | 5263(25.3) | 2797(14.2) | 1230(7.1) |  |
| **Comorbidity and medications** |  |  |  |  |  |
| T2DM (%) | 2031(9.3) | 682(3.3) | 390(2.0) | 314(1.8) | <0.001 |
| Hypertension (%) | 18193(83.7) | 15853(76.1) | 14061(71.3) | 11412(65.8) | <0.001 |
| Hyperlipidemia (%) | 20953(96.5) | 19653(94.3) | 18277(92.6) | 15782(90.9) | <0.001 |
| Ischemic heart disease (%) | 1125(5.2) | 769(3.7) | 590(3.0) | 441(2.5) | <0.001 |
| Heart failure (%) | 91(0.4) | 62(0.3) | 59(0.3) | 32(0.2) | 0.001 |
| Stroke (%) | 386(1.8) | 278(1.3) | 228(1.2) | 175(1.0) | <0.001 |
| Renal failure (%) | 438(2.0) | 304(1.5) | 264(1.3) | 175(1.0) | <0.001 |
| Antidiabetic drugs (%) | 1366(6.3) | 470(2.3) | 289(1.5) | 265(1.5) | <0.001 |
| Antihypertensive drugs (%) | 8609(39.6) | 6260(30.0) | 4873(24.7) | 3553(20.5) | <0.001 |
| Antihypertriglyceridemic drugs (%) | 128(0.6) | 78(0.4) | 58(0.3) | 45(0.3) | <0.001 |
| Antihypercholesterolemic drugs (%) | 6882(31.7) | 4833(23.2) | 3794(19.2) | 2800(16.1) | <0.001 |
| Steroids-related drug (%) | 1038(4.8) | 978(4.7) | 776(3.9) | 638(3.7) | <0.001 |
| **Liver diseases and liver status** |  |  |  |  |  |
| MASLD (%) | 10249(47.2) | 5063(24.3) | 2422(12.3) | 971(5.6) | <0.001 |
| MetALD (%) | 1714(7.9) | 838(4.0) | 335(1.7) | 123(0.7) | <0.001 |
| ALD (%) | 213(1.0) | 96(0.5) | 43(0.2) | 20(0.1) | <0.001 |
| Viral hepatitis (%) | 10(0.0) | 13(0.1) | 5(0.0) | 26(0.1) | <0.001 |
| ALT (U/L) | 24.63(13.22) | 20.91(12.00) | 19.45(11.14) | 18.49(10.36) | <0.001 |
| AST (U/L) | 25.95(9.36) | 25.07(8.52) | 25.09(9.15) | 25.50(8.90) | <0.001 |
| ALB (g/L) | 45.08(2.54) | 45.06(2.51) | 45.19(2.52) | 45.26(2.60) | <0.001 |
| TBIL (umol/L) | 7.89(3.31) | 8.17(3.54) | 8.31(3.58) | 8.48(3.70) | <0.001 |
| Uric (umol/L) | 314.35(69.96) | 283.83(62.16) | 265.47(58.81) | 248.40(55.67) | <0.001 |
| Crea (umol/L) | 65.53(15.44) | 65.05(12.61) | 64.29(11.61) | 63.45(14.93) | <0.001 |
| FIB4 | 1.28(1.22) | 1.39(2.06) | 1.43(0.53) | 1.54(1.05) | <0.001 |
| **Sex hormones** |  |  |  |  |  |
| SHBG (nmol/L) | 30.39(7.00) | 48.22(4.52) | 65.19(5.60) | 96.66(19.58) | <0.001 |
| TT (nmol/L) | 1.09(0.53) | 1.09(0.52) | 1.07(0.51) | 1.06(0.51) | <0.001 |
| E2 (pmol/L)* | 297.73(229.48) | 329.60(237.42) | 364.99(293.54) | 482.59(525.54) | <0.001 |
| **Follow-up outcomes** |  |  |  |  |  |
| Liver fibrosis and cirrhosis (%) | 101(0.5) | 71(0.3) | 87(0.4) | 86(0.5) | 0.101 |
| Hepatocellular cancer (%) | 10(0.0) | 7(0.0) | 8(0.0) | 13(0.1) | 0.285 |
| Liver related death (%) | 13(0.1) | 11(0.1) | 16(0.1) | 21(0.1) | 0.076 |

TDI: townsend deprivation index; T2DM: type 2 diabetes mellitus; MASLD: Metabolic dysfunction-associated steatotic liver disease; MetALD: Metabolic dysfunction-associated steatotic liver disease with increased alcohol intake; ALD: alcohol-related liver disease; ALT: alanine aminotransferase; AST: aspartate aminotransferase; ALB: albumin; TBIL: total bilirubin; Uric: uric acid; Crea: creatinine; FIB4: fibrosis-4 index; SHBG: sex hormone binding globulin; TT: total testosterone; E2: oestradiol.

Continuous variables (age, alcohol intake per day, ALT, AST, ALB, TBIL, Uric, Crea, FIB4, SHBG, TT and E2) are presented as means (standard deviations), and categorical variables are expressed as N (percentages). The t-test was employed to compare differences in continuous variables, while chi-square tests were used for categorical variables.

^#^Participants were categorized into quartiles based on sex hormone binding globulin levels.

*Among them, E2 was measured in 2,618 postmenopause.

Supplemental Table 4 Baseline characteristics of postmenopausal female population based on TT quartiles.

|  | Q1^#^ | Q2 | Q3 | Q4 |  |
| --- | --- | --- | --- | --- | --- |
| N | 22017 | 20589 | 19172 | 17859 |  |
| **‌Sociodemographic factors** |  |  |  |  |  |
| Age (years) | 60.73(5.24) | 60.45(5.33) | 60.23(5.41) | 60.14(5.53) | <0.001 |
| Race (white, %) | 21081(95.7) | 19668(95.5) | 18336(95.6) | 17090(95.7) | 0.716 |
| Qualifications (%) |  |  |  |  | <0.001 |
| College or University | 6565(29.8) | 6083(29.5) | 5591(29.2) | 5107(28.6) |  |
| A levels/AS levels | 2429(11.0) | 2265(11.0) | 2110(11.0) | 1893(10.6) |  |
| O levels/GCSEs | 5166(23.5) | 4907(23.8) | 4426(23.1) | 4184(23.4) |  |
| CSEs | 871(4.0) | 856(4.2) | 761(4.0) | 743(4.2) |  |
| NVQ or HND or HNC | 953(4.3) | 898(4.4) | 932(4.9) | 801(4.5) |  |
| Other professional qualifications | 1507(6.8) | 1381(6.7) | 1358(7.1) | 1156(6.5) |  |
| None of the above | 4526(20.6) | 4199(20.4) | 3994(20.8) | 3975(22.3) |  |
| TDI (%) |  |  |  |  | 0.063 |
| Tertile 1 | 7729(35.1) | 7220(35.1) | 6708(35.0) | 6038(33.8) |  |
| Tertile 2 | 7521(34.2) | 7064(34.3) | 6647(34.7) | 6192(34.7) |  |
| Tertile 3 | 6767(30.7) | 6305(30.6) | 5817(30.3) | 5629(31.5) |  |
| **Lifestyle factors** |  |  |  |  |  |
| Smoking status (%) |  |  |  |  | <0.001 |
| Never | 12886(58.5) | 11962(58.1) | 11307(59.0) | 10431(58.4) |  |
| Ever | 7639(34.7) | 7111(34.5) | 6250(32.6) | 5783(32.4) |  |
| Current | 1492(6.8) | 1516(7.4) | 1615(8.4) | 1645(9.2) |  |
| Alcohol intake per day (g) | 9.70(11.13) | 9.86(11.38) | 10.16(11.75) | 10.36(12.82) |  |
| Drinking status (%) |  |  |  |  | <0.001 |
| Never or special occasions | 6138(27.9) | 5536(26.9) | 5172(27.0) | 4992(28.0) |  |
| Monthly to weekly | 11799(53.6) | 11240(54.6) | 10261(53.5) | 9355(52.4) |  |
| Never or special occasions | 4080(18.5) | 3813(18.5) | 3739(19.5) | 3512(19.7) |  |
| Physical status (%) |  |  |  |  | <0.001 |
| Unhealthy | 11001(50.0) | 10367(50.4) | 9772(51.0) | 9206(51.5) |  |
| Healthy | 5306(24.1) | 4690(22.8) | 4307(22.5) | 3821(21.4) |  |
| Miss | 5710(25.9) | 5532(26.9) | 5093(26.6) | 4832(27.1) |  |
| Diet status (%) |  |  |  |  | <0.001 |
| Unhealthy | 15649(71.1) | 14861(72.2) | 13857(72.3) | 13181(73.8) |  |
| Healthy | 6187(28.1) | 5542(26.9) | 5147(26.8) | 4519(25.3) |  |
| Miss | 181(0.8) | 186(0.9) | 168(0.9) | 159(0.9) |  |
| Sleep status (%) |  |  |  |  | 0.092 |
| Unhealthy | 2963(13.5) | 2764(13.4) | 2611(13.6) | 2545(14.3) |  |
| Healthy | 18929(86.0) | 17694(85.9) | 16451(85.8) | 15189(85.0) |  |
| Miss | 125(0.6) | 131(0.6) | 110(0.6) | 125(0.7) |  |
| Sedentary status (%) |  |  |  |  | <0.001 |
| Unhealthy | 16786(76.2) | 15770(76.6) | 14820(77.3) | 13993(78.4) |  |
| Healthy | 5018(22.8) | 4599(22.3) | 4158(21.7) | 3650(20.4) |  |
| Miss | 213(1.0) | 220(1.1) | 194(1.0) | 216(1.2) |  |
| BMI status (%) |  |  |  |  | <0.001 |
| Underweight (<18.5) | 193(0.9) | 160(0.8) | 104(0.5) | 90(0.5) |  |
| Normal (18.5-24.99) | 8896(40.4) | 7776(37.8) | 6856(35.8) | 5552(31.1) |  |
| Overweight (25.0-29.99) | 8380(38.1) | 7956(38.6) | 7525(39.2) | 6907(38.7) |  |
| Obesity (>=30.0) | 4548(20.7) | 4697(22.8) | 4687(24.4) | 5310(29.7) |  |
| **Comorbidity and medications** |  |  |  |  |  |
| T2DM (%) | 961(4.4) | 872(4.2) | 745(3.9) | 839(4.7) | 0.002 |
| Hypertension (%) | 15975(72.6) | 15130(73.5) | 14413(75.2) | 14001(78.4) | <0.001 |
| Hyperlipidemia (%) | 20553(93.4) | 19299(93.7) | 17998(93.9) | 16815(94.2) | 0.009 |
| Ischemic heart disease (%) | 871(4.0) | 763(3.7) | 669(3.5) | 622(3.5) | 0.034 |
| Heart failure (%) | 62(0.3) | 64(0.3) | 60(0.3) | 58(0.3) | 0.878 |
| Stroke (%) | 338(1.5) | 257(1.2) | 226(1.2) | 246(1.4) | 0.009 |
| Renal failure (%) | 340(1.5) | 280(1.4) | 278(1.5) | 283(1.6) | 0.25 |
| Antidiabetic drugs (%) | 678(3.1) | 628(3.1) | 526(2.7) | 558(3.1) | 0.115 |
| Antihypertensive drugs (%) | 6253(28.4) | 5783(28.1) | 5561(29.0) | 5698(31.9) | <0.001 |
| Antihypertriglyceridemic drugs (%) | 96(0.4) | 80(0.4) | 69(0.4) | 64(0.4) | 0.55 |
| Antihypercholesterolemic drugs (%) | 5457(24.8) | 4774(23.2) | 4194(21.9) | 3884(21.7) | <0.001 |
| Steroids-related drug (%) | 1129(5.1) | 933(4.5) | 717(3.7) | 651(3.6) | <0.001 |
| **Liver diseases and liver status** |  |  |  |  |  |
| MASLD (%) | 4747(21.6) | 4649(22.6) | 4427(23.1) | 4882(27.3) | <0.001 |
| MetALD (%) | 757(3.4) | 729(3.5) | 753(3.9) | 771(4.3) | <0.001 |
| ALD (%) | 82(0.4) | 76(0.4) | 88(0.5) | 126(0.7) | <0.001 |
| Viral hepatitis (%) | 16(0.1) | 15(0.1) | 12(0.1) | 11(0.1) | 0.954 |
| ALT (U/L) | 20.65(10.96) | 20.75(12.68) | 21.05(11.40) | 21.84(13.15) | <0.001 |
| AST (U/L) | 25.35(8.20) | 25.25(9.02) | 25.32(8.16) | 25.75(10.62) | <0.001 |
| ALB (g/L) | 45.06(2.53) | 45.14(2.53) | 45.19(2.55) | 45.19(2.57) | <0.001 |
| TBIL (umol/L) | 7.99(3.48) | 8.12(3.50) | 8.29(3.54) | 8.45(3.62) | <0.001 |
| Uric (umol/L) | 274.27(65.62) | 276.51(65.63) | 281.07(66.23) | 289.44(69.44) | <0.001 |
| Crea (umol/L) | 64.65(11.83) | 64.40(11.30) | 64.57(13.65) | 65.01(18.01) | <0.001 |
| FIB4 | 1.41(0.65) | 1.39(0.51) | 1.40(1.33) | 1.41(2.34) | 0.303 |
| **Sex hormones** |  |  |  |  |  |
| SHBG (nmol/L) | 58.85(26.72) | 58.30(25.98) | 58.03(25.80) | 57.09(25.77) | <0.001 |
| TT (nmol/L) | 0.56(0.11) | 0.87(0.08) | 1.19(0.10) | 1.82(0.45) | <0.001 |
| E2 (pmol/L)* | 356.93(284.29) | 379.46(357.46) | 370.97(306.20) | 368.83(410.79) | 0.803 |
| **Follow-up outcomes** |  |  |  |  |  |
| Liver fibrosis and cirrhosis (%) | 101(0.5) | 66(0.3) | 75(0.4) | 103(0.6) | 0.001 |
| Hepatocellular cancer (%) | 12(0.1) | 8(0.0) | 3(0.0) | 15(0.1) | 0.022 |
| Liver related death (%) | 14(0.1) | 10(0.0) | 13(0.1) | 24(0.1) | 0.014 |

TDI: townsend deprivation index; T2DM: type 2 diabetes mellitus; MASLD: Metabolic dysfunction-associated steatotic liver disease; MetALD: Metabolic dysfunction-associated steatotic liver disease with increased alcohol intake; ALD: alcohol-related liver disease; ALT: alanine aminotransferase; AST: aspartate aminotransferase; ALB: albumin; TBIL: total bilirubin; Uric: uric acid; Crea: creatinine; FIB4: fibrosis-4 index; SHBG: sex hormone binding globulin; TT: total testosterone; E2: oestradiol.

Continuous variables (age, alcohol intake per day, ALT, AST, ALB, TBIL, Uric, Crea, FIB4, SHBG, TT and E2) are presented as means (standard deviations), and categorical variables are expressed as N (percentages). The t-test was employed to compare differences in continuous variables, while chi-square tests were used for categorical variables.

^#^Participants were categorized into quartiles based on total testosterone levels.

*Among them, E2 was measured in 2,618 postmenopause.

Supplemental Table 5 Baseline characteristics of premenopausal female population based on SHBG quartiles.

|  | Q1^#^ | Q2 | Q3 | Q4 |  |
| --- | --- | --- | --- | --- | --- |
| N | 5270 | 6149 | 7268 | 9487 |  |
| **‌Sociodemographic factors** |  |  |  |  |  |
| Age (years) | 46.03(4.54) | 45.85(4.22) | 45.84(4.09) | 45.81(3.96) | 0.012 |
| Race (white, %) | 4550(86.3) | 5565(90.5) | 6700(92.2) | 8837(93.1) | <0.001 |
| Qualifications (%) |  |  |  |  | <0.001 |
| College or University | 1983(37.6) | 2516(40.9) | 3239(44.6) | 4317(45.5) |  |
| A levels/AS levels | 758(14.4) | 888(14.4) | 1062(14.6) | 1479(15.6) |  |
| O levels/GCSEs | 1310(24.9) | 1451(23.6) | 1634(22.5) | 2075(21.9) |  |
| CSEs | 486(9.2) | 543(8.8) | 524(7.2) | 721(7.6) |  |
| NVQ or HND or HNC | 221(4.2) | 229(3.7) | 280(3.9) | 290(3.1) |  |
| Other professional qualifications | 172(3.3) | 209(3.4) | 237(3.3) | 281(3.0) |  |
| None of the above | 340(6.5) | 313(5.1) | 292(4.0) | 324(3.4) |  |
| TDI (%) |  |  |  |  | <0.001 |
| Tertile 1 | 1456(27.6) | 1844(30.0) | 2268(31.2) | 3023(31.9) |  |
| Tertile 2 | 1617(30.7) | 2030(33.0) | 2461(33.9) | 3070(32.4) |  |
| Tertile 3 | 2197(41.7) | 2275(37.0) | 2539(34.9) | 3394(35.8) |  |
| **Lifestyle factors** |  |  |  |  |  |
| Smoking status (%) |  |  |  |  | <0.001 |
| Never | 3421(64.9) | 3871(63.0) | 4632(63.7) | 6244(65.8) |  |
| Ever | 1305(24.8) | 1596(26.0) | 1837(25.3) | 2384(25.1) |  |
| Current | 544(10.3) | 682(11.1) | 799(11.0) | 859(9.1) |  |
| Alcohol intake per day (g) | 10.04(12.94) | 11.90(13.48) | 11.86(13.08) | 11.57(12.24) | <0.001 |
| Drinking status (%) |  |  |  |  | <0.001 |
| Never or special occasions | 1690(32.1) | 1397(22.7) | 1429(19.7) | 1872(19.7) |  |
| Monthly to weekly | 2934(55.7) | 3778(61.4) | 4631(63.7) | 6159(64.9) |  |
| Never or special occasions | 646(12.3) | 974(15.8) | 1208(16.6) | 1456(15.3) |  |
| Physical status (%) |  |  |  |  | <0.001 |
| Unhealthy | 3178(60.3) | 3701(60.2) | 4284(58.9) | 5423(57.2) |  |
| Healthy | 998(18.9) | 1299(21.1) | 1704(23.4) | 2471(26.0) |  |
| Miss | 1094(20.8) | 1149(18.7) | 1280(17.6) | 1593(16.8) |  |
| Diet status (%) |  |  |  |  | <0.001 |
| Unhealthy | 4431(84.1) | 5115(83.2) | 5901(81.2) | 7458(78.6) |  |
| Healthy | 792(15.0) | 989(16.1) | 1324(18.2) | 1974(20.8) |  |
| Miss | 47(0.9) | 45(0.7) | 43(0.6) | 55(0.6) |  |
| Sleep status (%) |  |  |  |  | <0.001 |
| Unhealthy | 645(12.2) | 698(11.4) | 705(9.7) | 948(10.0) |  |
| Healthy | 4588(87.1) | 5429(88.3) | 6538(90.0) | 8519(89.8) |  |
| Miss | 37(0.7) | 22(0.4) | 25(0.3) | 20(0.2) |  |
| Sedentary status (%) |  |  |  |  | <0.001 |
| Unhealthy | 3868(73.4) | 4198(68.3) | 4834(66.5) | 6106(64.4) |  |
| Healthy | 1357(25.7) | 1897(30.9) | 2385(32.8) | 3322(35.0) |  |
| Miss | 45(0.9) | 54(0.9) | 49(0.7) | 59(0.6) |  |
| BMI status (%) |  |  |  |  | <0.001 |
| Underweight (<18.5) | 5(0.1) | 15(0.2) | 52(0.7) | 144(1.5) |  |
| Normal (18.5-24.99) | 915(17.4) | 2241(36.4) | 3829(52.7) | 6386(67.3) |  |
| Overweight (25.0-29.99) | 1889(35.8) | 2402(39.1) | 2483(34.2) | 2366(24.9) |  |
| Obesity (>=30.0) | 2461(46.7) | 1491(24.2) | 904(12.4) | 591(6.2) |  |
| **Comorbidity and medications** |  |  |  |  |  |
| T2DM (%) | 267(5.1) | 90(1.5) | 76(1.0) | 79(0.8) | <0.001 |
| Hypertension (%) | 3148(59.7) | 2862(46.5) | 2826(38.9) | 3370(35.5) | <0.001 |
| Hyperlipidemia (%) | 4544(86.2) | 4566(74.3) | 4950(68.1) | 6127(64.6) | <0.001 |
| Ischemic heart disease (%) | 56(1.1) | 39(0.6) | 48(0.7) | 26(0.3) | <0.001 |
| Heart failure (%) | 4(0.1) | 2(0.0) | 2(0.0) | 6(0.1) | 0.539 |
| Stroke (%) | 37(0.7) | 37(0.6) | 41(0.6) | 31(0.3) | 0.01 |
| Renal failure (%) | 22(0.4) | 18(0.3) | 18(0.2) | 30(0.3) | 0.403 |
| Antidiabetic drugs (%) | 197(3.7) | 71(1.2) | 61(0.8) | 70(0.7) | <0.001 |
| Antihypertensive drugs (%) | 895(17.0) | 657(10.7) | 558(7.7) | 606(6.4) | <0.001 |
| Antihypertriglyceridemic drugs (%) | 8(0.2) | 10(0.2) | 3(0.0) | 2(0.0) | 0.003 |
| Antihypercholesterolemic drugs (%) | 568(10.8) | 331(5.4) | 337(4.6) | 422(4.4) | <0.001 |
| Steroids-related drug (%) | 190(3.6) | 177(2.9) | 224(3.1) | 231(2.4) | 0.001 |
| **Liver diseases and liver status** |  |  |  |  |  |
| MASLD (%) | 1981(37.6) | 962(15.6) | 500(6.9) | 272(2.9) | <0.001 |
| MetALD (%) | 354(6.7) | 237(3.9) | 133(1.8) | 78(0.8) | <0.001 |
| ALD (%) | 57(1.1) | 42(0.7) | 31(0.4) | 21(0.2) | <0.001 |
| Viral hepatitis (%) | 1(0.0) | 3(0.0) | 2(0.0) | 9(0.1) | 0.159 |
| ALT (U/L) | 20.43(12.12) | 17.41(9.93) | 16.18(8.44) | 15.85(10.18) | <0.001 |
| AST (U/L) | 22.43(7.25) | 21.66(7.42) | 21.58(7.37) | 21.90(8.95) | <0.001 |
| ALB (g/L) | 44.76(2.59) | 44.90(2.51) | 45.02(2.57) | 45.04(2.64) | <0.001 |
| TBIL (umol/L) | 7.87(3.66) | 8.42(4.17) | 8.64(4.17) | 8.82(4.47) | <0.001 |
| Uric (umol/L) | 282.66(63.47) | 255.82(55.59) | 241.47(52.64) | 231.47(50.19) | <0.001 |
| Crea (umol/L) | 63.26(10.12) | 63.42(9.45) | 63.40(11.01) | 62.96(11.05) | 0.017 |
| FIB4 | 0.89(0.46) | 1.05(7.46) | 1.00(0.36) | 1.05(0.49) | 0.039 |
| **Sex hormones** |  |  |  |  |  |
| SHBG (nmol/L) | 30.59(7.07) | 48.35(4.53) | 65.66(5.60) | 102.00(23.83) | <0.001 |
| TT (nmol/L) | 1.25(0.56) | 1.23(0.54) | 1.20(0.51) | 1.17(0.52) | <0.001 |
| E2 (pmol/L)* | 461.48(299.71) | 512.74(335.85) | 556.49(385.43) | 678.89(554.67) | <0.001 |
| **Follow-up outcomes** |  |  |  |  |  |
| Liver fibrosis and cirrhosis (%) | 11(0.2) | 19(0.3) | 13(0.2) | 25(0.3) | 0.428 |
| Hepatocellular cancer (%) | 0(0.0) | 1(0.0) | 0(0.0) | 3(0.0) | 0.281 |
| Liver related death (%) | 2(0.0) | 3(0.0) | 3(0.0) | 5(0.1) | 0.976 |

TDI: townsend deprivation index; T2DM: type 2 diabetes mellitus; MASLD: Metabolic dysfunction-associated steatotic liver disease; MetALD: Metabolic dysfunction-associated steatotic liver disease with increased alcohol intake; ALD: alcohol-related liver disease; ALT: alanine aminotransferase; AST: aspartate aminotransferase; ALB: albumin; TBIL: total bilirubin; Uric: uric acid; Crea: creatinine; FIB4: fibrosis-4 index; SHBG: sex hormone binding globulin; TT: total testosterone; E2: oestradiol.

Continuous variables (age, alcohol intake per day, ALT, AST, ALB, TBIL, Uric, Crea, FIB4, SHBG, TT and E2) are presented as means (standard deviations), and categorical variables are expressed as N (percentages). The t-test was employed to compare differences in continuous variables, while chi-square tests were used for categorical variables.

^#^Participants were categorized into quartiles based on sex hormone binding globulin levels.

*Among them, E2 was measured in 19,924 premenopause.

Supplemental Table 6 Baseline characteristics of premenopausal female population based on TT quartiles.

|  | Q1^#^ | Q2 | Q3 | Q4 |  |
| --- | --- | --- | --- | --- | --- |
| N | 5000 | 6443 | 7850 | 8881 |  |
| **‌Sociodemographic factors** |  |  |  |  |  |
| Age (years) | 46.33(4.44) | 46.10(4.28) | 45.75(4.08) | 45.54(3.96) | <0.001 |
| Race (white, %) | 4473(89.5) | 5816(90.3) | 7166(91.3) | 8197(92.3) | <0.001 |
| Qualifications (%) |  |  |  |  | 0.117 |
| College or University | 2155(43.1) | 2799(43.4) | 3392(43.2) | 3709(41.8) |  |
| A levels/AS levels | 743(14.9) | 924(14.3) | 1190(15.2) | 1330(15.0) |  |
| O levels/GCSEs | 1156(23.1) | 1495(23.2) | 1749(22.3) | 2070(23.3) |  |
| CSEs | 353(7.1) | 520(8.1) | 660(8.4) | 741(8.3) |  |
| NVQ or HND or HNC | 191(3.8) | 213(3.3) | 299(3.8) | 317(3.6) |  |
| Other professional qualifications | 161(3.2) | 210(3.3) | 239(3.0) | 289(3.3) |  |
| None of the above | 241(4.8) | 282(4.4) | 321(4.1) | 425(4.8) |  |
| TDI (%) |  |  |  |  | 0.065 |
| Tertile 1 | 1575(31.5) | 1955(30.3) | 2436(31.0) | 2625(29.6) |  |
| Tertile 2 | 1609(32.2) | 2119(32.9) | 2584(32.9) | 2866(32.3) |  |
| Tertile 3 | 1816(36.3) | 2369(36.8) | 2830(36.1) | 3390(38.2) |  |
| **Lifestyle factors** |  |  |  |  |  |
| Smoking status (%) |  |  |  |  | <0.001 |
| Never | 3292(65.8) | 4189(65.0) | 5084(64.8) | 5603(63.1) |  |
| Ever | 1330(26.6) | 1677(26.0) | 1971(25.1) | 2144(24.1) |  |
| Current | 378(7.6) | 577(9.0) | 795(10.1) | 1134(12.8) |  |
| Alcohol intake per day (g) | 10.34(11.53) | 10.81(12.05) | 11.26(12.24) | 12.64(14.54) | <0.001 |
| Drinking status (%) |  |  |  |  | <0.001 |
| Never or special occasions | 1229(24.6) | 1467(22.8) | 1738(22.1) | 1954(22.0) |  |
| Monthly to weekly | 3096(61.9) | 4073(63.2) | 4946(63.0) | 5387(60.7) |  |
| Never or special occasions | 675(13.5) | 903(14.0) | 1166(14.9) | 1540(17.3) |  |
| Physical status (%) |  |  |  |  | 0.052 |
| Unhealthy | 2871(57.4) | 3791(58.8) | 4651(59.2) | 5273(59.4) |  |
| Healthy | 1219(24.4) | 1496(23.2) | 1804(23.0) | 1953(22.0) |  |
| Miss | 910(18.2) | 1156(17.9) | 1395(17.8) | 1655(18.6) |  |
| Diet status (%) |  |  |  |  | 0.561 |
| Unhealthy | 4027(80.5) | 5230(81.2) | 6369(81.1) | 7279(82.0) |  |
| Healthy | 938(18.8) | 1169(18.1) | 1430(18.2) | 1542(17.4) |  |
| Miss | 35(0.7) | 44(0.7) | 51(0.6) | 60(0.7) |  |
| Sleep status (%) |  |  |  |  | 0.613 |
| Unhealthy | 512(10.2) | 674(10.5) | 838(10.7) | 972(10.9) |  |
| Healthy | 4468(89.4) | 5748(89.2) | 6988(89.0) | 7870(88.6) |  |
| Miss | 20(0.4) | 21(0.3) | 24(0.3) | 39(0.4) |  |
| Sedentary status (%) |  |  |  |  | 0.003 |
| Unhealthy | 3336(66.7) | 4255(66.0) | 5271(67.1) | 6144(69.2) |  |
| Healthy | 1628(32.6) | 2140(33.2) | 2521(32.1) | 2672(30.1) |  |
| Miss | 36(0.7) | 48(0.7) | 58(0.7) | 65(0.7) |  |
| BMI status (%) |  |  |  |  | <0.001 |
| Underweight (<18.5) | 51(1.0) | 61(0.9) | 55(0.7) | 49(0.6) |  |
| Normal (18.5-24.99) | 2586(51.7) | 3202(49.7) | 3856(49.1) | 3727(42.0) |  |
| Overweight (25.0-29.99) | 1533(30.7) | 2043(31.7) | 2472(31.5) | 3092(34.8) |  |
| Obesity (>=30.0) | 830(16.6) | 1137(17.6) | 1467(18.7) | 2013(22.7) |  |
| **Comorbidity and medications** |  |  |  |  |  |
| T2DM (%) | 103(2.1) | 119(1.8) | 134(1.7) | 156(1.8) | 0.493 |
| Hypertension (%) | 2051(41.0) | 2718(42.2) | 3246(41.4) | 4191(47.2) | <0.001 |
| Hyperlipidemia (%) | 3603(72.1) | 4603(71.4) | 5576(71.0) | 6405(72.1) | 0.392 |
| Ischemic heart disease (%) | 40(0.8) | 41(0.6) | 42(0.5) | 46(0.5) | 0.168 |
| Heart failure (%) | 2(0.0) | 7(0.1) | 2(0.0) | 3(0.0) | 0.112 |
| Stroke (%) | 28(0.6) | 33(0.5) | 31(0.4) | 54(0.6) | 0.275 |
| Renal failure (%) | 22(0.4) | 21(0.3) | 12(0.2) | 33(0.4) | 0.018 |
| Antidiabetic drugs (%) | 81(1.6) | 88(1.4) | 100(1.3) | 130(1.5) | 0.409 |
| Antihypertensive drugs (%) | 475(9.5) | 612(9.5) | 695(8.9) | 934(10.5) | 0.003 |
| Antihypertriglyceridemic drugs (%) | 5(0.1) | 1(0.0) | 11(0.1) | 6(0.1) | 0.067 |
| Antihypercholesterolemic drugs (%) | 333(6.7) | 376(5.8) | 446(5.7) | 503(5.7) | 0.078 |
| Steroids-related drug (%) | 162(3.2) | 181(2.8) | 223(2.8) | 256(2.9) | 0.512 |
| **Liver diseases and liver status** |  |  |  |  |  |
| MASLD (%) | 593(11.9) | 813(12.6) | 965(12.3) | 1344(15.1) | <0.001 |
| MetALD (%) | 94(1.9) | 169(2.6) | 218(2.8) | 321(3.6) | <0.001 |
| ALD (%) | 19(0.4) | 27(0.4) | 39(0.5) | 66(0.7) | 0.01 |
| Viral hepatitis (%) | 3(0.1) | 5(0.1) | 2(0.0) | 5(0.1) | 0.59 |
| ALT (U/L) | 16.71(9.08) | 16.90(9.06) | 16.73(9.49) | 17.89(12.13) | <0.001 |
| AST (U/L) | 21.77(6.55) | 21.80(6.83) | 21.64(7.36) | 22.15(9.68) | <0.001 |
| ALB (g/L) | 44.77(2.56) | 44.86(2.60) | 44.99(2.58) | 45.09(2.58) | <0.001 |
| TBIL (umol/L) | 8.19(4.11) | 8.33(4.02) | 8.55(4.20) | 8.78(4.35) | <0.001 |
| Uric (umol/L) | 243.29(57.09) | 245.64(56.61) | 247.10(56.29) | 256.14(59.39) | <0.001 |
| Crea (umol/L) | 63.58(9.77) | 63.39(9.77) | 63.24(9.40) | 62.90(12.28) | 0.001 |
| FIB4 | 1.01(0.35) | 1.00(0.44) | 0.98(0.45) | 1.03(6.22) | 0.844 |
| **Sex hormones** |  |  |  |  |  |
| SHBG (nmol/L) | 70.01(32.52) | 68.76(31.39) | 67.79(30.15) | 65.09(29.80) | <0.001 |
| TT (nmol/L) | 0.57(0.10) | 0.88(0.08) | 1.20(0.10) | 1.82(0.43) | <0.001 |
| E2 (pmol/L)* | 499.91(368.95) | 542.13(414.62) | 574.06(443.48) | 631.57(478.23) | <0.001 |
| **Follow-up outcomes** |  |  |  |  |  |
| Liver fibrosis and cirrhosis (%) | 10(0.2) | 12(0.2) | 17(0.2) | 29(0.3) | 0.257 |
| Hepatocellular cancer (%) | 0(0.0) | 1(0.0) | 3(0.0) | 0(0.0) | 0.16 |
| Liver related death (%) | 1(0.0) | 4(0.1) | 5(0.1) | 3(0.0) | 0.59 |

TDI: townsend deprivation index; T2DM: type 2 diabetes mellitus; MASLD: Metabolic dysfunction-associated steatotic liver disease; MetALD: Metabolic dysfunction-associated steatotic liver disease with increased alcohol intake; ALD: alcohol-related liver disease; ALT: alanine aminotransferase; AST: aspartate aminotransferase; ALB: albumin; TBIL: total bilirubin; Uric: uric acid; Crea: creatinine; FIB4: fibrosis-4 index; SHBG: sex hormone binding globulin; TT: total testosterone; E2: oestradiol.

Continuous variables (age, alcohol intake per day, ALT, AST, ALB, TBIL, Uric, Crea, FIB4, SHBG, TT and E2) are presented as means (standard deviations), and categorical variables are expressed as N (percentages). The t-test was employed to compare differences in continuous variables, while chi-square tests were used for categorical variables.

^#^Participants were categorized into quartiles based on total testosterone levels.

*Among them, E2 was measured in 19,924 premenopause.

Supplemental table 7 Testing the proportional hazards assumption for Cox regression models.

| **Group** | **Exposure** | **Outcome** | **Chisq** | **P** |
| --- | --- | --- | --- | --- |
| Male | SHBG group | Liver fibrosis and cirrhosis | 25.03 | <0.001 |
| Male | SHBG group | Hepatocellular cancer | 1.74 | 0.628 |
| Male | SHBG group | Liver related death | 2.83 | 0.418 |
| Male | TT group | Liver fibrosis and cirrhosis | 6.64 | 0.084 |
| Male | TT group | Hepatocellular cancer | 1.29 | 0.731 |
| Male | TT group | Liver related death | 1.08 | 0.782 |
| Postmenopause | SHBG group | Liver fibrosis and cirrhosis | 17.71 | 0.001 |
| Postmenopause | SHBG group | Hepatocellular cancer | 3.38 | 0.336 |
| Postmenopause | SHBG group | Liver related death | 4.81 | 0.186 |
| Postmenopause | TT group | Liver fibrosis and cirrhosis | 2.87 | 0.413 |
| Postmenopause | TT group | Hepatocellular cancer | 4.37 | 0.224 |
| Postmenopause | TT group | Liver related death | 0.52 | 0.915 |

SHBG: sex hormone-binding globulin; TT: total testosterone.

Supplemental Table 8 Time-stratified sensitivity analyses for associations between SHBG and liver fibrosis and cirrhosis.

|  | Followup: 0-10 years | | | Followup: >10 years | | |
| --- | --- | --- | --- | --- | --- | --- |
|  | N (%) | HR (95%CI) | P | N (%) | HR (95%CI) | P |
| Male | | | | | | |
| Q1 | 118(0.30) | Ref |  | 99(0.27) | Ref |  |
| Q2 | 149(0.38) | 1.51(1.18-1.93) | 0.001 | 111(0.3) | 1.34(1.02-1.77) | 0.036 |
| Q3 | 151(0.39) | 1.64(1.28-2.10) | <0.001 | 120(0.33) | 1.65(1.25-2.18) | <0.001 |
| Q4 | 379(0.98) | 4.05(3.22-5.08) | <0.001 | 188(0.53) | 2.95(2.25-3.86) | <0.001 |
| Postmenopause | | | | | | |
| Q1 | 43(0.2) | Ref |  | 58(0.28) | Ref |  |
| Q2 | 39(0.19) | 1.72(1.1-2.69) | 0.017 | 32(0.16) | 0.95(0.61-1.5) | 0.838 |
| Q3 | 56(0.28) | 3.49(2.27-5.36) | <0.001 | 31(0.16) | 1.24(0.77-2.01) | 0.379 |
| Q4 | 60(0.35) | 6.23(3.98-9.76) | <0.001 | 26(0.16) | 1.55(0.9-2.68) | 0.114 |

Adjusted for age, race, qualifications, townsend deprivation index, smoking status, alcohol intake frequency, alcohol intake per day, BMI status, physical activity, diet status, sleep status, and sedentary status, ALT, AST, ALB, TBIL, Uric, Crea, FIB4, comorbidity (hypertension, diabetes, hyperlipidemia, ischemic heart disease, heart failure, stroke, renal failure) and medications (antihypertensive drugs, antidiabetic drugs, antihypertriglyceridemic drugs, antihypercholesterolemic drugs, steroids-related drug), baseline liver diseases status (MASLD, MetALD, ALD, viral hepatitis).

HR: hazard ratios; CI: confidence interval; SHBG: sex hormone-binding globulin; TT: total testosterone; Q1: quartile 1; Q2: quartile 2; Q3: quartile 3; Q4: quartile 4; Ref: reference; BMI: body mass index.

Supplemental Table 9 Fine-Gray competing risks analyses for liver-related death.

|  | Male | | | Postmenopause | | |
| --- | --- | --- | --- | --- | --- | --- |
|  | N (%) | HR (95%CI) | P | N (%) | HR (95%CI) | P |
| SHBG | | | | | | |
| Q1 | 49(0.13) | Ref |  | 13(0.06) | Ref |  |
| Q2 | 59(0.15) | 1.39(0.94-2.06) | 0.097 | 11(0.05) | 1.76(0.74-4.16) | 0.200 |
| Q3 | 67(0.17) | 1.68(1.14-2.47) | 0.009 | 16(0.08) | 3.67(1.59-8.47) | 0.002 |
| Q4 | 218(0.56) | 5.07(3.54-7.26) | <0.001 | 21(0.12) | 8.46(3.67-19.5) | <0.001 |
| TT | | | | | | |
| Q1 | 90(0.23) | Ref |  | 14(0.06) | Ref |  |
| Q2 | 74(0.19) | 1.11(0.81-1.52) | 0.510 | 10(0.05) | 0.72(0.32-1.64) | 0.440 |
| Q3 | 78(0.2) | 1.29(0.92-1.79) | 0.140 | 13(0.07) | 0.91(0.42-1.98) | 0.820 |
| Q4 | 151(0.39) | 2.51(1.86-3.39) | <0.001 | 24(0.13) | 1.32(0.65-2.7) | 0.440 |

Adjusted for age, race, qualifications, townsend deprivation index, smoking status, alcohol intake frequency, alcohol intake per day, BMI status, physical activity, diet status, sleep status, and sedentary status, ALT, AST, ALB, TBIL, Uric, Crea, FIB4, comorbidity (hypertension, diabetes, hyperlipidemia, ischemic heart disease, heart failure, stroke, renal failure) and medications (antihypertensive drugs, antidiabetic drugs, antihypertriglyceridemic drugs, antihypercholesterolemic drugs, steroids-related drug), baseline liver diseases status (MASLD, MetALD, ALD, viral hepatitis).

HR: hazard ratios; CI: confidence interval; SHBG: sex hormone-binding globulin; TT: total testosterone; Q1: quartile 1; Q2: quartile 2; Q3: quartile 3; Q4: quartile 4; Ref: reference; BMI: body mass index.

Supplemental Table 10 Sensitivity analyses with mutual adjustment of SHBG and TT in Cox regression models for CLD-related outcomes.

|  |  | Male | | | Postmenopause | | |
| --- | --- | --- | --- | --- | --- | --- | --- |
|  |  | N (%) | HR (95%CI) | P | N (%) | HR (95%CI) | P |
| **Liver fibrosis and cirrhosis** | | | | | | | |
| SHBG | Q1 | 217(0.56) | Ref |  | 101(0.46) | Ref |  |
|  | Q2 | 260(0.67) | 1.42(1.17-1.71) | <0.001 | 71(0.34) | 1.31(0.96-1.79) | 0.092 |
|  | Q3 | 271(0.69) | 1.58(1.3-1.93) | <0.001 | 87(0.44) | 2.28(1.67-3.11) | <0.001 |
|  | Q4 | 567(1.46) | 3.27(2.67-4.01) | <0.001 | 86(0.5) | 3.54(2.53-4.96) | <0.001 |
| **Hepatocellular cancer** | | | | | | | |
| SHBG | Q1 | 29(0.07) | Ref |  | 10(0.05) | Ref |  |
|  | Q2 | 22(0.06) | 0.91(0.52-1.6) | 0.744 | 7(0.03) | 1.16(0.51-2.64) | 0.72 |
|  | Q3 | 42(0.11) | 1.83(1.09-3.08) | 0.022 | 8(0.04) | 1.63(0.75-3.56) | 0.22 |
|  | Q4 | 119(0.31) | 4.91(2.92-8.26) | <0.001 | 13(0.07) | 3.64(1.86-7.12) | <0.001 |
| **Liver related death** | | | | | | | |
| SHBG | Q1 | 49(1.58) | Ref |  | 13(0.7) | Ref |  |
|  | Q2 | 59(1.57) | 1.2(0.81-1.78) | 0.356 | 11(0.67) | 1.02(0.52-1.99) | 0.952 |
|  | Q3 | 67(1.55) | 1.39(0.93-2.08) | 0.108 | 16(1.06) | 2.19(1.23-3.9) | 0.008 |
|  | Q4 | 218(3.88) | 3.28(2.21-4.86) | <0.001 | 21(1.47) | 4.71(2.76-8.05) | <0.001 |
| **Liver fibrosis and cirrhosis** | | | | | | | |
| TT | Q1 | 316(0.81) | Ref |  | 101(0.46) | Ref |  |
|  | Q2 | 271(0.69) | 0.93(0.78-1.1) | 0.39 | 66(0.32) | 0.62(0.45-0.85) | 0.003 |
|  | Q3 | 312(0.8) | 1(0.84-1.2) | 0.994 | 75(0.39) | 0.8(0.59-1.08) | 0.149 |
|  | Q4 | 416(1.07) | 1.19(0.98-1.43) | 0.075 | 103(0.58) | 0.92(0.69-1.22) | 0.558 |
| **Hepatocellular cancer** | | | | | | | |
| TT | Q1 | 58(0.15) | Ref |  | 12(0.05) | Ref |  |
|  | Q2 | 35(0.09) | 0.61(0.39-0.96) | 0.031 | 8(0.04) | 0.58(0.27-1.27) | 0.177 |
|  | Q3 | 50(0.13) | 0.74(0.48-1.16) | 0.188 | 3(0.02) | 0.27(0.08-0.88) | 0.029 |
|  | Q4 | 69(0.18) | 0.84(0.53-1.33) | 0.467 | 15(0.08) | 1.26(0.66-2.42) | 0.486 |
| **Liver related death** | | | | | | | |
| TT | Q1 | 90(1.84) | Ref |  | 14(0.78) | Ref |  |
|  | Q2 | 74(1.81) | 0.81(0.58-1.13) | 0.216 | 10(0.64) | 0.7(0.36-1.39) | 0.312 |
|  | Q3 | 78(2.01) | 0.83(0.59-1.18) | 0.306 | 13(0.85) | 0.92(0.5-1.71) | 0.802 |
|  | Q4 | 151(3.84) | 1(0.71-1.41) | 1.000 | 24(1.55) | 0.82(0.49-1.39) | 0.467 |

Adjusted for race, qualifications, townsend deprivation index, smoking status, alcohol intake frequency, alcohol intake per day, BMI status, physical activity, diet status, sleep status, and sedentary status, ALT, AST, ALB, TBIL, Uric, Crea, FIB4, comorbidity (hypertension, diabetes, hyperlipidemia, ischemic heart disease, heart failure, stroke, renal failure) and medications (antihypertensive drugs, antidiabetic drugs, antihypertriglyceridemic drugs, antihypercholesterolemic drugs, steroids-related drug), baseline liver diseases status (MASLD, MetALD, ALD, viral hepatitis). Additionally, analyses of SHBG were further adjusted for TT, and analyses of TT were further adjusted for SHBG.

HR: hazard ratios; CI: confidence interval; SHBG: sex hormone-binding globulin; TT: total testosterone; Q1: quartile 1; Q2: quartile 2; Q3: quartile 3; Q4: quartile 4; Ref: reference; BMI: body mass index.

Supplemental Table 11 Cox analysis of sex hormone and chronic liver disease based on age subgroup in male population.

|  |  | Young (age < 65) | | | Old (age >= 65) | | |
| --- | --- | --- | --- | --- | --- | --- | --- |
|  |  | N (%) | HR (95%CI) | P | N (%) | HR (95%CI) | P |
| **Liver fibrosis and cirrhosis** | | | | | | | |
| SHBG | Q1 | 173(0.50) | Ref |  | 44(1.05) | Ref |  |
|  | Q2 | 208(0.65) | 1.56(1.28-1.92) | <0.001 | 52(0.76) | 1.00(0.67-1.50) | 0.995 |
|  | Q3 | 205(0.69) | 1.84(1.49-2.26) | <0.001 | 66(0.72) | 1.01(0.68-1.50) | 0.946 |
|  | Q4 | 386(1.44) | 3.82(3.14-4.64) | <0.001 | 181(1.52) | 2.68(1.89-3.80) | <0.001 |
| **Hepatocellular cancer** | | | | | | | |
| SHBG | Q1 | 19(0.05) | Ref |  | 10(0.24) | Ref |  |
|  | Q2 | 11(0.03) | 0.79(0.38-1.67) | 0.544 | 11(0.16) | 1.03(0.43-2.44) | 0.954 |
|  | Q3 | 25(0.08) | 2.09(1.14-3.84) | 0.018 | 17(0.19) | 1.30(0.57-2.92) | 0.531 |
|  | Q4 | 68(0.25) | 5.95(3.44-10.28) | <0.001 | 51(0.43) | 3.99(1.95-8.14) | <0.001 |
| **Liver related death** | | | | | | | |
| SHBG | Q1 | 35(0.10) | Ref |  | 14(0.33) | Ref |  |
|  | Q2 | 44(0.14) | 1.23(0.78-1.93) | 0.369 | 15(0.22) | 0.92(0.53-1.59) | 0.762 |
|  | Q3 | 47(0.16) | 1.40(0.90-2.20) | 0.136 | 20(0.22) | 1.09(0.67-1.77) | 0.724 |
|  | Q4 | 157(0.58) | 3.25(2.19-4.81) | <0.001 | 61(0.51) | 2.41(1.65-3.51) | <0.001 |
| **Liver fibrosis and cirrhosis** | | | | | | | |
| TT | Q1 | 215(0.71) | Ref |  | 101(1.13) | Ref |  |
|  | Q2 | 201(0.65) | 1.19(0.98-1.45) | 0.076 | 70(0.86) | 1.08(0.79-1.47) | 0.644 |
|  | Q3 | 234(0.75) | 1.53(1.26-1.85) | <0.001 | 78(0.99) | 1.37(1.00-1.87) | 0.048 |
|  | Q4 | 322(1.02) | 2.15(1.78-2.60) | <0.001 | 94(1.31) | 2.23(1.64-3.03) | <0.001 |
| **Hepatocellular cancer** | | | | | | | |
| TT | Q1 | 30(0.10) | Ref |  | 28(0.31) | Ref |  |
|  | Q2 | 20(0.06) | 0.89(0.50-1.59) | 0.699 | 15(0.18) | 0.85(0.45-1.63) | 0.634 |
|  | Q3 | 29(0.09) | 1.44(0.84-2.46) | 0.179 | 21(0.27) | 1.54(0.85-2.80) | 0.155 |
|  | Q4 | 44(0.14) | 2.09(1.25-3.50) | 0.005 | 25(0.35) | 2.44(1.34-4.42) | 0.003 |
| **Liver related death** | | | | | | | |
| TT | Q1 | 54(0.18) | Ref |  | 36(0.40) | Ref |  |
|  | Q2 | 49(0.16) | 0.99(0.67-1.48) | 0.974 | 25(0.31) | 1.06(0.68-1.66) | 0.791 |
|  | Q3 | 56(0.18) | 1.39(0.94-2.06) | 0.094 | 22(0.28) | 1.21(0.76-1.93) | 0.425 |
|  | Q4 | 124(0.39) | 2.29(1.61-3.25) | <0.001 | 27(0.38) | 1.51(0.98-2.33) | 0.063 |

Adjusted for race, qualifications, townsend deprivation index, smoking status, alcohol intake frequency, alcohol intake per day, BMI status, physical activity, diet status, sleep status, and sedentary status, ALT, AST, ALB, TBIL, Uric, Crea, FIB4, comorbidity (hypertension, diabetes, hyperlipidemia, ischemic heart disease, heart failure, stroke, renal failure) and medications (antihypertensive drugs, antidiabetic drugs, antihypertriglyceridemic drugs, antihypercholesterolemic drugs, steroids-related drug), baseline liver diseases status (MASLD, MetALD, ALD, viral hepatitis).

HR: hazard ratios; CI: confidence interval; SHBG: sex hormone-binding globulin; TT: total testosterone; Q1: quartile 1; Q2: quartile 2; Q3: quartile 3; Q4: quartile 4; Ref: reference; BMI: body mass index.

Supplemental Table 12 Cox analysis of sex hormone and chronic liver disease based on BMI subgroup in male population.

|  |  | Normal weight (18.5 <= BMI < 25) | | | Overweight or obesity (BMI >= 25) | | |
| --- | --- | --- | --- | --- | --- | --- | --- |
|  |  | N (%) | HR (95%CI) | P | N (%) | HR (95%CI) | P |
| **Liver fibrosis and cirrhosis** | | | | | | | |
| SHBG | Q1 | 11(0.24) | Ref |  | 206(0.60) | Ref |  |
|  | Q2 | 28(0.36) | 1.86(0.92-3.77) | 0.085 | 232(0.74) | 1.40(1.16-1.69) | <0.001 |
|  | Q3 | 45(0.42) | 2.18(1.11-4.28) | 0.024 | 226(0.80) | 1.55(1.27-1.88) | <0.001 |
|  | Q4 | 131(0.83) | 3.87(2.02-7.42) | <0.001 | 433(1.90) | 3.48(2.90-4.18) | <0.001 |
| **Hepatocellular cancer** | | | | | | | |
| SHBG | Q1 | 1(0.02) | Ref |  | 28(0.08) | Ref |  |
|  | Q2 | 0(0.00) | 0.00(0.00-Inf) | 0.891 | 22(0.07) | 0.87(0.50-1.53) | 0.632 |
|  | Q3 | 4(0.04) | 1.45(0.50-4.22) | 0.497 | 38(0.13) | 1.54(0.93-2.54) | 0.092 |
|  | Q4 | 21(0.13) | 3.92(1.47-10.46) | 0.006 | 98(0.43) | 4.30(2.74-6.76) | <0.001 |
| **Liver related death** | | | | | | | |
| SHBG | Q1 | 6(0.13) | Ref |  | 43(0.12) | Ref |  |
|  | Q2 | 6(0.08) | 0.41(0.12-1.38) | 0.152 | 53(0.17) | 1.20(0.79-1.80) | 0.395 |
|  | Q3 | 9(0.08) | 0.41(0.13-1.29) | 0.127 | 58(0.21) | 1.35(0.90-2.02) | 0.149 |
|  | Q4 | 48(0.30) | 0.90(0.35-2.35) | 0.834 | 169(0.74) | 3.44(2.40-4.93) | <0.001 |
| **Liver fibrosis and cirrhosis** | | | | | | | |
| TT | Q1 | 27(0.52) | Ref |  | 288(0.85) | Ref |  |
|  | Q2 | 29(0.36) | 0.68(0.40-1.17) | 0.168 | 242(0.78) | 1.17(0.98-1.39) | 0.075 |
|  | Q3 | 49(0.45) | 0.99(0.61-1.60) | 0.974 | 262(0.93) | 1.44(1.21-1.71) | <0.001 |
|  | Q4 | 110(0.75) | 1.57(1.01-2.45) | 0.044 | 305(1.27) | 2.10(1.77-2.50) | <0.001 |
| **Hepatocellular cancer** | | | | | | | |
| TT | Q1 | 3(0.06) | Ref |  | 55(0.16) | Ref |  |
|  | Q2 | 3(0.04) | 0.67(0.20-2.24) | 0.515 | 32(0.10) | 0.87(0.56-1.35) | 0.537 |
|  | Q3 | 6(0.05) | 1.45(0.58-3.63) | 0.423 | 44(0.16) | 1.36(0.90-2.07) | 0.145 |
|  | Q4 | 14(0.10) | 1.42(0.65-3.08) | 0.379 | 55(0.23) | 2.33(1.56-3.48) | <0.001 |
| **Liver related death** | | | | | | | |
| TT | Q1 | 10(0.19) | Ref |  | 80(0.24) | Ref |  |
|  | Q2 | 8(0.10) | 0.26(0.12-0.55) | <0.001 | 66(0.21) | 1.13(0.81-1.58) | 0.469 |
|  | Q3 | 12(0.11) | 0.42(0.22-0.78) | 0.006 | 66(0.24) | 1.42(1.01-2.00) | 0.041 |
|  | Q4 | 39(0.27) | 0.91(0.56-1.46) | 0.687 | 111(0.46) | 2.06(1.51-2.82) | <0.001 |

Adjusted for age, race, qualifications, townsend deprivation index, smoking status, alcohol intake frequency, alcohol intake per day, physical activity, diet status, sleep status, and sedentary status, ALT, AST, ALB, TBIL, Uric, Crea, FIB4, comorbidity (hypertension, diabetes, hyperlipidemia, ischemic heart disease, heart failure, stroke, renal failure) and medications (antihypertensive drugs, antidiabetic drugs, antihypertriglyceridemic drugs, antihypercholesterolemic drugs, steroids-related drug), baseline liver diseases status (MASLD, MetALD, ALD, viral hepatitis).

HR: hazard ratios; CI: confidence interval; SHBG: sex hormone-binding globulin; TT: total testosterone; Q1: quartile 1; Q2: quartile 2; Q3: quartile 3; Q4: quartile 4; Ref: reference; BMI: body mass index.

Supplemental Table 13 Cox analysis of sex hormone and chronic liver disease based on baseline liver diseases status in male population.

|  |  | Baseline without liver diseases | | | Baseline with liver diseases | | |
| --- | --- | --- | --- | --- | --- | --- | --- |
|  |  | N (%) | HR (95%CI) | P | N (%) | HR (95%CI) | P |
| **Liver fibrosis and cirrhosis** | | | | | | | |
| SHBG | Q1 | 13(0.13) | Ref |  | 204(0.71) | Ref |  |
|  | Q2 | 45(0.28) | 2.06(1.11-3.83) | 0.023 | 215(0.94) | 1.35(1.11-1.64) | 0.003 |
|  | Q3 | 61(0.30) | 2.05(1.11-3.77) | 0.021 | 210(1.13) | 1.57(1.29-1.92) | <0.001 |
|  | Q4 | 136(0.53) | 3.14(1.74-5.68) | <0.001 | 431(3.30) | 3.88(3.23-4.66) | <0.001 |
| **Hepatocellular cancer** | | | | | | | |
| SHBG | Q1 | 1(0.01) | Ref |  | 28(0.10) | Ref |  |
|  | Q2 | 3(0.02) | 1.79(0.54-5.87) | 0.337 | 19(0.08) | 0.79(0.44-1.42) | 0.424 |
|  | Q3 | 6(0.03) | 2.55(1.05-6.19) | 0.039 | 36(0.19) | 1.62(0.98-2.69) | 0.061 |
|  | Q4 | 22(0.09) | 6.47(3.07-13.68) | <0.001 | 97(0.74) | 4.78(3.04-7.52) | <0.001 |
| **Liver related death** | | | | | | | |
| SHBG | Q1 | 3(0.03) | Ref |  | 46(0.16) | Ref |  |
|  | Q2 | 8(0.05) | 1.59(0.76-3.35) | 0.219 | 51(0.22) | 1.07(0.72-1.61) | 0.727 |
|  | Q3 | 10(0.05) | 1.01(0.51-1.99) | 0.981 | 57(0.31) | 1.31(0.88-1.95) | 0.184 |
|  | Q4 | 41(0.16) | 3.20(1.89-5.42) | <0.001 | 177(1.36) | 3.24(2.28-4.62) | <0.001 |
| **Liver fibrosis and cirrhosis** | | | | | | | |
| TT | Q1 | 34(0.32) | Ref |  | 282(1.00) | Ref |  |
|  | Q2 | 37(0.23) | 0.70(0.43-1.12) | 0.139 | 234(1.02) | 1.18(0.99-1.41) | 0.059 |
|  | Q3 | 73(0.36) | 1.16(0.77-1.76) | 0.469 | 239(1.28) | 1.44(1.21-1.72) | <0.001 |
|  | Q4 | 111(0.44) | 1.33(0.89-1.97) | 0.162 | 305(2.27) | 2.37(1.99-2.81) | <0.001 |
| **Hepatocellular cancer** | | | | | | | |
| TT | Q1 | 6(0.06) | Ref |  | 52(0.18) | Ref |  |
|  | Q2 | 4(0.02) | 0.46(0.16-1.31) | 0.144 | 31(0.14) | 0.94(0.60-1.47) | 0.773 |
|  | Q3 | 9(0.04) | 0.95(0.44-2.06) | 0.901 | 41(0.22) | 1.46(0.95-2.24) | 0.08 |
|  | Q4 | 13(0.05) | 1.17(0.58-2.36) | 0.667 | 56(0.42) | 2.59(1.72-3.89) | <0.001 |
| **Liver related death** | | | | | | | |
| TT | Q1 | 7(0.06) | Ref |  | 83(0.29) | Ref |  |
|  | Q2 | 11(0.07) | 0.80(0.42-1.54) | 0.505 | 63(0.27) | 1.03(0.73-1.44) | 0.872 |
|  | Q3 | 13(0.06) | 0.81(0.44-1.50) | 0.509 | 65(0.35) | 1.36(0.97-1.90) | 0.075 |
|  | Q4 | 31(0.12) | 1.49(0.90-2.45) | 0.12 | 120(0.89) | 2.17(1.59-2.96) | <0.001 |

Adjusted for age, race, qualifications, townsend deprivation index, smoking status, alcohol intake frequency, alcohol intake per day, BMI status, physical activity, diet status, sleep status, and sedentary status, ALT, AST, ALB, TBIL, Uric, Crea, FIB4, comorbidity (hypertension, diabetes, hyperlipidemia, ischemic heart disease, heart failure, stroke, renal failure) and medications (antihypertensive drugs, antidiabetic drugs, antihypertriglyceridemic drugs, antihypercholesterolemic drugs, steroids-related drug).

HR: hazard ratios; CI: confidence interval; SHBG: sex hormone-binding globulin; TT: total testosterone; Q1: quartile 1; Q2: quartile 2; Q3: quartile 3; Q4: quartile 4; Ref: reference; BMI: body mass index.

Supplemental Table 14 Cox analysis of sex hormone and chronic liver disease based on age subgroup in postmenopausal female population.

|  |  | Young (age < 65) | | | Old (age >= 65) | | |
| --- | --- | --- | --- | --- | --- | --- | --- |
|  |  | N (%) | HR (95%CI) | P | N (%) | HR (95%CI) | P |
| **Liver fibrosis and cirrhosis** | | | | | | | |
| SHBG | Q1 | 72(0.43) | Ref |  | 29(0.59) | Ref |  |
|  | Q2 | 50(0.33) | 1.49(1.03-2.17) | 0.036 | 21(0.38) | 0.84(0.52-1.37) | 0.49 |
|  | Q3 | 63(0.43) | 2.58(1.77-3.75) | <0.001 | 24(0.47) | 1.66(1.05-2.63) | 0.03 |
|  | Q4 | 60(0.48) | 3.92(2.61-5.91) | <0.001 | 26(0.55) | 2.62(1.67-4.09) | <0.001 |
| **Hepatocellular cancer** | | | | | | | |
| SHBG | Q1 | 4(0.02) | Ref |  | 6(0.12) | Ref |  |
|  | Q2 | 5(0.03) | 2.08(0.77-5.59) | 0.149 | 2(0.04) | 0.55(0.12-2.43) | 0.428 |
|  | Q3 | 5(0.03) | 2.80(1.04-7.54) | 0.042 | 3(0.06) | 0.84(0.23-3.01) | 0.787 |
|  | Q4 | 9(0.07) | 7.05(3.05-16.30) | <0.001 | 4(0.08) | 2.08(0.66-6.56) | 0.209 |
| **Liver related death** | | | | | | | |
| SHBG | Q1 | 9(0.05) | Ref |  | 4(0.08) | Ref |  |
|  | Q2 | 9(0.06) | 1.35(0.63-2.89) | 0.445 | 2(0.04) | 0.41(0.10-1.79) | 0.237 |
|  | Q3 | 11(0.08) | 2.81(1.40-5.64) | 0.004 | 5(0.10) | 2.20(0.77-6.26) | 0.14 |
|  | Q4 | 13(0.10) | 4.05(2.08-7.88) | <0.001 | 8(0.17) | 7.13(2.80-18.16) | <0.001 |
| **Liver fibrosis and cirrhosis** | | | | | | | |
| TT | Q1 | 72(0.45) | Ref |  | 29(0.49) | Ref |  |
|  | Q2 | 43(0.28) | 0.57(0.39-0.84) | 0.005 | 23(0.44) | 0.87(0.55-1.39) | 0.571 |
|  | Q3 | 55(0.38) | 0.80(0.56-1.14) | 0.218 | 20(0.42) | 0.80(0.49-1.30) | 0.37 |
|  | Q4 | 75(0.56) | 0.91(0.65-1.27) | 0.586 | 28(0.63) | 1.22(0.79-1.89) | 0.372 |
| **Hepatocellular cancer** | | | | | | | |
| TT | Q1 | 8(0.05) | Ref |  | 4(0.07) | Ref |  |
|  | Q2 | 2(0.01) | 0.15(0.04-0.64) | 0.01 | 6(0.11) | 1.51(0.54-4.26) | 0.436 |
|  | Q3 | 3(0.02) | 0.31(0.09-1.03) | 0.057 | 0(0.00) | 0.00(0.00-Inf) | 0.964 |
|  | Q4 | 10(0.07) | 1.13(0.49-2.58) | 0.772 | 5(0.11) | 1.84(0.63-5.39) | 0.267 |
| **Liver related death** | | | | | | | |
| TT | Q1 | 10(0.06) | Ref |  | 4(0.07) | Ref |  |
|  | Q2 | 6(0.04) | 0.73(0.30-1.73) | 0.471 | 4(0.08) | 0.80(0.26-2.48) | 0.7 |
|  | Q3 | 9(0.06) | 0.90(0.43-1.88) | 0.769 | 4(0.08) | 1.14(0.37-3.50) | 0.82 |
|  | Q4 | 17(0.13) | 0.87(0.46-1.65) | 0.678 | 7(0.16) | 3.43(1.31-8.97) | 0.012 |

Adjusted for race, qualifications, townsend deprivation index, smoking status, alcohol intake frequency, alcohol intake per day, BMI status, physical activity, diet status, sleep status, and sedentary status, ALT, AST, ALB, TBIL, Uric, Crea, FIB4, comorbidity (hypertension, diabetes, hyperlipidemia, ischemic heart disease, heart failure, stroke, renal failure) and medications (antihypertensive drugs, antidiabetic drugs, antihypertriglyceridemic drugs, antihypercholesterolemic drugs, steroids-related drug), baseline liver diseases status (MASLD, MetALD, ALD, viral hepatitis).

HR: hazard ratios; CI: confidence interval; SHBG: sex hormone-binding globulin; TT: total testosterone; Q1: quartile 1; Q2: quartile 2; Q3: quartile 3; Q4: quartile 4; Ref: reference; BMI: body mass index.

Supplemental Table 15 Cox analysis of sex hormone and chronic liver disease based on BMI subgroup in postmenopausal female population.

|  |  | Normal weight (18.5 <= BMI < 25) | | | Overweight or obesity (BMI >= 25) | | |
| --- | --- | --- | --- | --- | --- | --- | --- |
|  |  | N (%) | HR (95%CI) | P | N (%) | HR (95%CI) | P |
| **Liver fibrosis and cirrhosis** | | | | | | | |
| SHBG | Q1 | 7(0.23) | Ref |  | 94(0.50) | Ref |  |
|  | Q2 | 8(0.13) | 0.76(0.36-1.59) | 0.47 | 62(0.43) | 1.34(0.96-1.87) | 0.082 |
|  | Q3 | 28(0.31) | 2.43(1.51-3.93) | <0.001 | 58(0.55) | 1.90(1.33-2.71) | <0.001 |
|  | Q4 | 27(0.26) | 2.44(1.51-3.96) | <0.001 | 58(0.91) | 4.13(2.87-5.95) | <0.001 |
| **Hepatocellular cancer** | | | | | | | |
| SHBG | Q1 | 0(0.00) | - | - | 10(0.05) | Ref |  |
|  | Q2 | 1(0.02) | - | - | 6(0.04) | 1.11(0.45-2.73) | 0.823 |
|  | Q3 | 3(0.03) | - | - | 5(0.05) | 1.15(0.44-3.03) | 0.775 |
|  | Q4 | 6(0.06) | - | - | 7(0.11) | 3.25(1.38-7.65) | 0.007 |
| **Liver related death** | | | | | | | |
| SHBG | Q1 | 1(0.03) | Ref |  | 12(0.06) | Ref |  |
|  | Q2 | 2(0.03) | 20.51(4.2-101.4) | <0.001 | 9(0.06) | 1.06(0.40-2.81) | 0.899 |
|  | Q3 | 3(0.03) | 1.11(0.28-4.44) | 0.887 | 13(0.12) | 2.95(1.21-7.22) | 0.018 |
|  | Q4 | 6(0.06) | 5.14(1.49-17.81) | 0.01 | 15(0.23) | 4.84(1.83-12.79) | 0.001 |
| **Liver fibrosis and cirrhosis** | | | | | | | |
| TT | Q1 | 21(0.24) | Ref |  | 80(0.62) | Ref |  |
|  | Q2 | 17(0.22) | 0.90(0.52-1.55) | 0.694 | 47(0.37) | 0.50(0.34-0.73) | <0.001 |
|  | Q3 | 14(0.20) | 0.91(0.50-1.63) | 0.743 | 61(0.50) | 0.75(0.54-1.05) | 0.098 |
|  | Q4 | 18(0.32) | 1.03(0.60-1.76) | 0.923 | 84(0.69) | 0.89(0.65-1.21) | 0.455 |
| **Hepatocellular cancer** | | | | | | | |
| TT | Q1 | 4(0.04) | Ref |  | 8(0.06) | Ref |  |
|  | Q2 | 4(0.05) | 0.65(0.18-2.42) | 0.523 | 4(0.03) | 0.46(0.16-1.33) | 0.152 |
|  | Q3 | 0(0.00) | 0.00(0.00-Inf) | 0.984 | 3(0.02) | 0.34(0.10-1.12) | 0.075 |
|  | Q4 | 2(0.04) | 0.53(0.11-2.50) | 0.419 | 13(0.11) | 1.67(0.80-3.52) | 0.174 |
| **Liver related death** | | | | | | | |
| TT | Q1 | 3(0.03) | Ref |  | 11(0.09) | Ref |  |
|  | Q2 | 1(0.01) | 0.02(0.00-0.21) | 0.001 | 9(0.07) | 0.86(0.34-2.22) | 0.76 |
|  | Q3 | 5(0.07) | 8.28(2.35-29.12) | <0.001 | 8(0.07) | 0.65(0.24-1.73) | 0.387 |
|  | Q4 | 3(0.05) | 4.18(0.87-20.12) | 0.074 | 21(0.17) | 1.18(0.51-2.73) | 0.696 |

Adjusted for age, race, qualifications, townsend deprivation index, smoking status, alcohol intake frequency, alcohol intake per day, physical activity, diet status, sleep status, and sedentary status, ALT, AST, ALB, TBIL, Uric, Crea, FIB4, comorbidity (hypertension, diabetes, hyperlipidemia, ischemic heart disease, heart failure, stroke, renal failure) and medications (antihypertensive drugs, antidiabetic drugs, antihypertriglyceridemic drugs, antihypercholesterolemic drugs, steroids-related drug), baseline liver diseases status (MASLD, MetALD, ALD, viral hepatitis).

HR: hazard ratios; CI: confidence interval; SHBG: sex hormone-binding globulin; TT: total testosterone; Q1: quartile 1; Q2: quartile 2; Q3: quartile 3; Q4: quartile 4; Ref: reference; BMI: body mass index.

Supplemental Table 16 Cox analysis of sex hormone and chronic liver disease based on baseline liver diseases status in postmenopausal female population.

|  |  | Baseline without liver diseases | | | Baseline with liver diseases | | |
| --- | --- | --- | --- | --- | --- | --- | --- |
|  |  | N (%) | HR (95%CI) | P | N (%) | HR (95%CI) | P |
| **Liver fibrosis and cirrhosis** | | | | | | | |
| SHBG | Q1 | 21(0.22) | Ref |  | 80(0.66) | Ref |  |
|  | Q2 | 23(0.16) | 0.82(0.45-1.50) | 0.522 | 48(0.80) | 1.34(0.92-1.94) | 0.124 |
|  | Q3 | 42(0.25) | 1.38(0.80-2.39) | 0.247 | 45(1.61) | 2.38(1.61-3.52) | <0.001 |
|  | Q4 | 38(0.23) | 1.37(0.77-2.44) | 0.281 | 48(4.23) | 6.46(4.39-9.50) | <0.001 |
| **Hepatocellular cancer** | | | | | | | |
| SHBG | Q1 | 1(0.01) | Ref |  | 9(0.07) | Ref |  |
|  | Q2 | 2(0.01) | 2.59(0.58-11.47) | 0.211 | 5(0.08) | 0.88(0.33-2.38) | 0.805 |
|  | Q3 | 6(0.04) | 7.12(2.52-20.10) | <0.001 | 2(0.07) | 0.47(0.11-2.03) | 0.315 |
|  | Q4 | 6(0.04) | 8.40(2.98-23.65) | <0.001 | 7(0.62) | 4.87(2.00-11.85) | <0.001 |
| **Liver related death** | | | | | | | |
| SHBG | Q1 | 2(0.02) | Ref |  | 11(0.09) | Ref |  |
|  | Q2 | 3(0.02) | 1.34(0.38-4.74) | 0.651 | 8(0.13) | 0.99(0.35-2.79) | 0.982 |
|  | Q3 | 4(0.02) | 0.66(0.21-2.12) | 0.49 | 12(0.43) | 3.37(1.31-8.70) | 0.012 |
|  | Q4 | 7(0.04) | 1.99(0.71-5.63) | 0.193 | 14(1.23) | 6.05(2.24-16.37) | <0.001 |
| **Liver fibrosis and cirrhosis** | | | | | | | |
| TT | Q1 | 43(0.26) | Ref |  | 58(1.04) | Ref |  |
|  | Q2 | 25(0.17) | 0.60(0.37-1.00) | 0.048 | 41(0.75) | 0.68(0.45-1.04) | 0.078 |
|  | Q3 | 30(0.22) | 0.81(0.50-1.29) | 0.37 | 45(0.85) | 0.85(0.57-1.28) | 0.442 |
|  | Q4 | 26(0.22) | 0.68(0.41-1.12) | 0.133 | 77(1.33) | 1.18(0.82-1.68) | 0.372 |
| **Hepatocellular cancer** | | | | | | | |
| TT | Q1 | 7(0.04) | Ref |  | 5(0.09) | Ref |  |
|  | Q2 | 4(0.03) | 0.46(0.15-1.47) | 0.191 | 4(0.07) | 0.71(0.24-2.10) | 0.541 |
|  | Q3 | 0(0.00) | 0.00(0.00-Inf) | 0.987 | 3(0.06) | 0.58(0.17-1.94) | 0.373 |
|  | Q4 | 4(0.03) | 0.68(0.22-2.15) | 0.515 | 11(0.19) | 2.34(1.03-5.30) | 0.042 |
| **Liver related death** | | | | | | | |
| TT | Q1 | 5(0.03) | Ref |  | 9(0.16) | Ref |  |
|  | Q2 | 1(0.01) | 0.23(0.03-1.79) | 0.162 | 9(0.16) | 0.95(0.35-2.60) | 0.918 |
|  | Q3 | 6(0.04) | 3.02(1.09-8.38) | 0.034 | 7(0.13) | 0.63(0.21-1.88) | 0.41 |
|  | Q4 | 4(0.03) | 1.64(0.52-5.11) | 0.396 | 20(0.35) | 1.21(0.48-3.04) | 0.684 |

Adjusted for age, race, qualifications, townsend deprivation index, smoking status, alcohol intake frequency, alcohol intake per day, BMI status, physical activity, diet status, sleep status, and sedentary status, ALT, AST, ALB, TBIL, Uric, Crea, FIB4, comorbidity (hypertension, diabetes, hyperlipidemia, ischemic heart disease, heart failure, stroke, renal failure) and medications (antihypertensive drugs, antidiabetic drugs, antihypertriglyceridemic drugs, antihypercholesterolemic drugs, steroids-related drug).

HR: hazard ratios; CI: confidence interval; SHBG: sex hormone-binding globulin; TT: total testosterone; Q1: quartile 1; Q2: quartile 2; Q3: quartile 3; Q4: quartile 4; Ref: reference; BMI: body mass index.

Supplemental Figure 1 Cox analyses of associations between sex hormones and chronic liver disease-related outcomes in premenopausal females.

Adjusted for age, race, qualifications, townsend deprivation index, smoking status, alcohol intake frequency, alcohol intake per day, BMI status, physical activity, diet status, sleep status, and sedentary status, ALT, AST, ALB, TBIL, Uric, Crea, FIB4, comorbidity (hypertension, diabetes, hyperlipidemia, ischemic heart disease, heart failure, stroke, renal failure) and medications (antihypertensive drugs, antidiabetic drugs, antihypertriglyceridemic drugs, antihypercholesterolemic drugs, steroids-related drug), baseline liver diseases status (MASLD, MetALD, ALD, viral hepatitis).

HR: hazard ratios; CI: confidence interval; SHBG: sex hormone-binding globulin; TT: total testosterone; Low: lower than median; High: higher than median; Ref: reference; BMI: body mass index.
